# Strong and Fatigue‐Resistant Hydrogels via Poor Solvent Evaporation Assisted Hot‐Stretching for Tendon Repair

**DOI:** 10.1002/advs.202503697

**Published:** 2025-05-08

**Authors:** Huamin Li, Ying Zhang, Haidi Wu, Zhanqi Liu, Cheng Guan, Jin Zhang, Jingyi Chen, Shaohua He, Xuewu Huang, Wancheng Gu, Yiu Wing Mai, Jiefeng Gao

**Affiliations:** ^1^ School of Chemistry and Chemical Engineering Yangzhou University No 180, Road Siwangting Yangzhou Jiangsu 225002 P. R. China; ^2^ MOE Key Laboratory for Analytical Science of Food Safety and Biology College of Chemistry Fuzhou University 2 Xueyuan Road Fuzhou 350108 P. R. China; ^3^ College of Chemical Engineering Fuzhou University 2 Xueyuan Road Fuzhou 350108 P. R. China; ^4^ Qingyuan Innovation Laboratory 1 Xueyuan Road Quanzhou 362801 P. R. China; ^5^ Fuzhou University Affiliated Provincial Hospital 134 Dongjie Road Fuzhou Fujian 350001 P. R. China; ^6^ Department of Mechanical Engineering The Hong Kong Polytechnic University Hung Hom Kowloon Hong Kong 999077 P. R. China

**Keywords:** anisotropic hydrogels, flaw‐insensitivity, hot‐stretching, poor solvent evaporation, tendon repair

## Abstract

It is highly desirable but still remains challenging to develop high‐performance hydrogels with satisfactory mechanical properties for tissue engineering. Here, anisotropic yet transparent hydrogels (AHs) are prepared for tendon repair via a facile “poor solvent evaporation assisted hot‐stretching” strategy. AHs have great mechanical properties with tensile strength, toughness, and fracture energy as high as 33.14 ± 2.05 MPa, 44.1 ± 3.5 MJ m^−3^, and 106.18 ± 7.2 kJ m^−2^, respectively. Especially, AHs show unique flaw‐insensitive characteristics, and cracks can only deflect along the fiber alignment direction rather than propagate transverse to this direction, showing an interesting self‐protection function. The high strength, toughness, and fatigue resistance originate from the hierarchal structure of AHs, i.e., the densified polymeric network comprising fiber bundles and nanofibrils with aligned macromolecular chains, crystalline domains, and intermolecular hydrogen bonds. AHs with superior biocompatibility and swelling resistance can be used to repair rat tendons, and implantation of AHs can promote collagen regeneration for the tendon repair. This study provides a new method to fabricate strong and anti‐fatigue hydrogels as a new class of promising materials for soft tissues.

## Graphical abstract

An anisotropic hydrogel (AHs) is prepared in this study. The strength of AHs can reach up to 33.14 ± 2.05 MPa, and it exhibits unique defect‐insensitive characteristics. Cracks can only deflect along the fiber alignment direction, demonstrating an interesting self‐protection function. Due to its excellent biocompatibility, it can promote the repair of damaged tendons in rats.

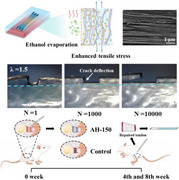

## 1 Introduction

Hydrogels with high water content,^[^ ^1^, ^2^ ^]^ flexibility, and biocompatibility resemble native extracellular matrix (ECM) in both structure and composition,^[^ ^3^, ^4^ ^]^ and have been promising candidates for artificial soft tissue materials.^[^ ^5^, ^6^, ^7^ ^]^ But their weak mechanical properties, e.g., low tensile strength and poor fatigue resistance, become one of the largest obstacles limiting their practical applications.^[^ ^8^, ^9^, ^10^ ^]^ Inspired by the well‐ordered and fibrous structures of natural tissues (e.g., tendons and ligaments),^[^ ^11^, ^12^, ^13^ ^]^ several strategies have been developed to induce macromolecular chain alignment for preparation of anisotropic hydrogels with enhanced mechanical properties.^[^ ^14^, ^15^, ^16^, ^17^ ^]^ Unidirectional freezing can cause directional growth of ice, and macromolecules are expelled and confined between adjacent ice crystals, forming anisotropic structures in hydrogels.^[^ ^18^, ^19^ ^]^ However, the enhancement in mechanical properties for the ice‐templated hydrogels is not satisfactory. Therefore, ions are introduced to the hydrogels to further improve the mechanical performance by “salting out” or coordination effect.^[^ ^20^, ^21^ ^]^ Multi‐scale reinforcement, such as fibril bridging and pinning by crystalline domains, provides high toughness and fracture energy. An issue is that these ions readily migrate out of the hydrogels if they are for in vivo use, which not only leads to large reductions in mechanical properties but also cause cell dehydration and rupture. Mechanical stretching has also been widely adopted to align macromolecules to fabricate anisotropic hydrogels (AHs).^[^ ^22^, ^23^, ^24^ ^]^ Hence, Mredha et al.^[^ ^25^ ^]^ proposed a drying in confined condition (DCC) method to produce strong hydrogels with aligned multiscale fibrous structures. But this strategy is only applicable for rigid polymers, such as alginate and cellulose, and no anisotropic structure was obtained for polyvinyl alcohol (PVA) gels. Mechanical training, i.e., cyclic stretching of the hydrogels in a water bath, could create aligned nanofibrillar architectures, e.g., skeletal muscles; and mechanically trained hydrogels with high water content displayed fatigue‐resistant performance.^[^ ^26^ ^]^ Nanofillers, such as nano ‐fibers, were also incorporated into the hydrogels to further improve the strength and elastic modulus of AHs.^[^ ^27^, ^28^, ^29^, ^30^ ^]^ Despite these significant advances, it is still challenging to develop anisotropic hydrogels as implantable soft issues having simultaneously improved tensile strength, fracture energy, and crack propagation resistance while maintaining good stretchability.

Herein, we propose a facile “poor solvent evaporation assisted hot‐stretching” strategy to prepare anisotropic yet transparent hydrogels with aligned multiscale fiber structures. This work introduces the synergistic effect of poor solvent evaporation and wet annealing for the preparation of anisotropic hydrogels (AHs). The high‐boiling‐point solvent glycerol ensures the thermal stability and structural stability of the organogel during the annealing process; the low‐boiling‐point ethanol volatilizes during the thermal stretching process, enhancing the stress in the stretching direction, promoting the orientation and crystallization of macromolecular chains, and facilitating the formation of neatly arranged nanofibrils. Importantly, our method avoids the use of ions, which improves the biocompatibility of the hydrogels. The tensile strength and fracture energy of the resulting hydrogels can reach as high as 33.14 ± 2.05 MPa and 106.18 ± 7.2 kJ m^−2^, respectively, with a satisfactory fracture strain of 113.67 ± 8.5%. Moreover, the hydrogels exhibit outstanding fatigue resistance, and cracks can only deflect along the fiber orientation direction rather than transverse to the fibrous structure, exhibiting a unique self‐protection function in the structure. Because of their superior mechanical properties, biocompatibility, and swelling resistance, AHs can be utilized as implantable artificial tendons, and the aligned fibrous structure can also provide anisotropic biophysical signals and further modulate the cell growth performance. In addition, aligned fibrils can enhance tendon gene expression, and promote the formation of parallel collagen fibers and tendon‐like tissues.^[^ ^31^ ^]^

## 2 Results and Discussion

### 2.1 Design of Anisotropic Hydrogels (AHs)

**Figure 1:**
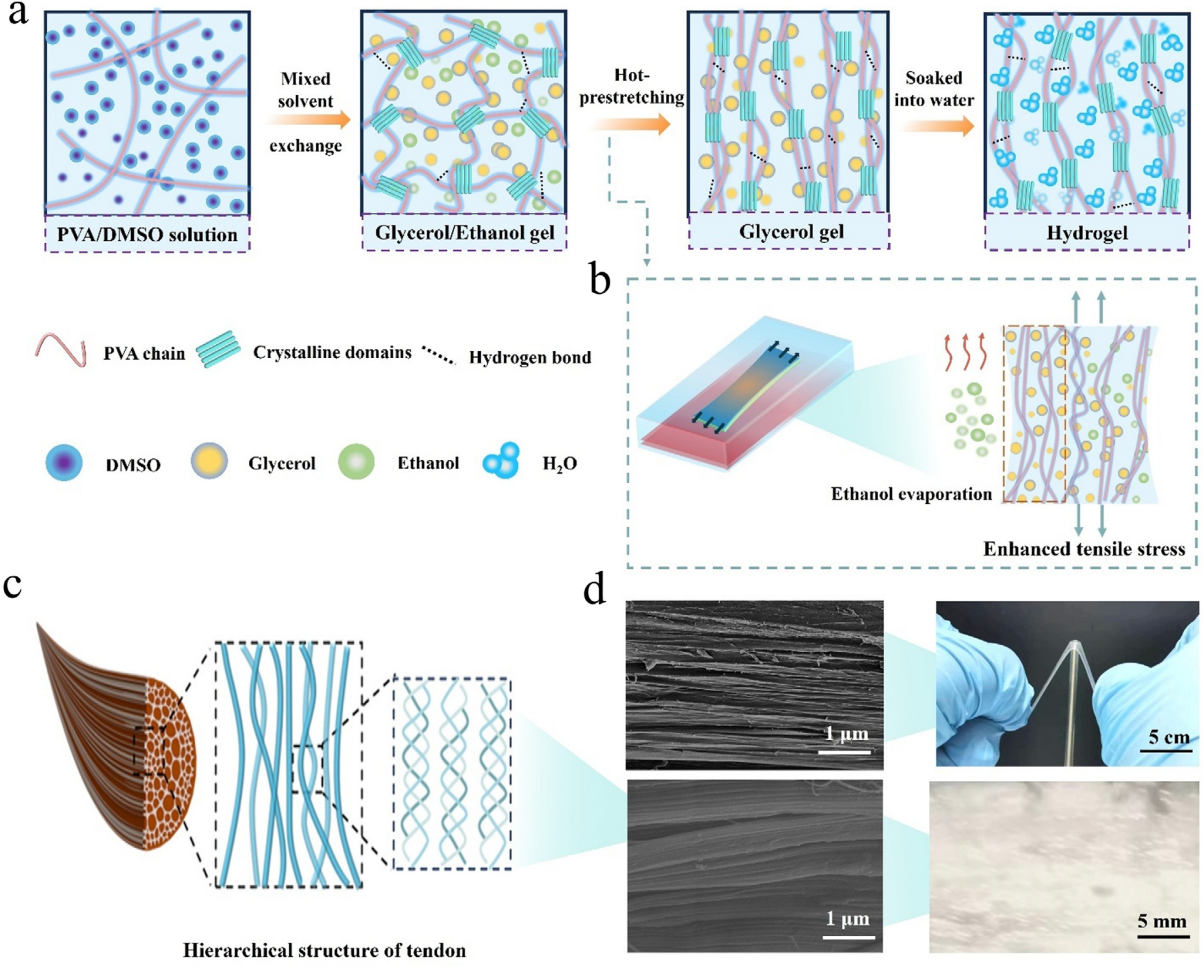
Bioinspired design and structure of anisotropic polyvinyl alcohol (PVA) hydrogels (AHs). a) Schematic for the preparation of AHs by poor solvent evaporation assisted hot‐stretching. b) Poor solvent evaporation promoted macromolecular chain aggregation and alignment. c) Structures of natural tendon. d) SEM images and photographs of AH‐200 and tendon.

**Figure** **1** illustrates details of the strategy, i.e., poor solvent evaporation assisted hot‐stretching, for preparation of highly transparent and strong anisotropic PVA hydrogels (AHs) with comprehensive properties matching those of tendons (Figure 1a). In brief, a homogeneous PVA/DMSO solution was first formed, and dissolution of PVA in its good solvent (DMSO) disentangled the macro‐molecules and created extended macromolecular chain conformations and pre‐assemblies. Afterward, DMSO was replaced by a mixture of glycerol/ethanol, both of which are poor solvents of PVA. This solvent exchange process caused macromolecular chain aggregation and further formation of crystal regions. In contrast, the abundance of hydroxyl groups in the PVA chains easily forms hydrogen bonds with glycerol and ethanol, yielding a 3D network. In this way, the polymer solution was transformed to PVA organogel. Then, the organogel was pre‐stretched to a certain ratio and subjected to wet‐annealing. Figure 1b describes the process of ethanol evaporation induced macromolecule alignment. Ethanol was rapidly evaporated, and the gel shrank to some degree in the thickness and width directions, producing sufficient extra tensile stresses in the stretch direction. The low‐boiling‐point solvent (ethanol) volatilizes during the thermal stretching of the organogel, thereby enhancing the axial stress along the stretching direction. This process promotes macromolecular chain alignment and crystallization, facilitating the formation of well‐ordered nanofibrils that significantly improve the material's mechanical properties. Meanwhile, the high‐boiling‐point solvent (glycerol) ensures thermal and structural stability of the organogel during stretching. It modulates macromolecular conformations through enhanced intermolecular interactions, enabling structural densification and increased crystallinity. The hydrogels with different glycerol/ethanol ratios and pre‐stretching ratios (PSRs) are designated, hereafter, HGxEy‐Z, where x: y is the mass ratio of glycerol to ethanol, and Z% the PSR applied during wet‐annealing. The isotropic PVA hydrogel is denoted IH. HG_4_E_1_‐Z are used for characterization and performance tests and are represented by AH‐Z. The pre‐stretching or PVA chain alignment direction is defined as the parallel direction, while that perpendicular to the pre‐stretching refers to the transverse direction. During wet‐annealing, the weight of the organogel decreased continuously until complete evaporation of ethanol. For example, it took 35 min for the organogel with a ratio of glycerol to ethanol of 4:1 to complete the evaporation, and then the mass of the PVA/glycerol kept unchanged because of the high boiling point of glycerol, hence the thermal stability of the organogel (Figure S1, Supporting Information). To further investigate this process, we performed stress relaxation experiments during the dynamic evaporation of ethanol. As shown in Figure S2 (Supporting Information), both the glycerol gel and the glycerol/ethanol gel exhibited solid‐like relaxation behavior. After 1800 s, the residual stress values were 1.44 and 2.59 MPa, respectively. In the initial phase of relaxation, the density of hydrogen bonds decreases sharply, resulting in a rapid increase in the mobility of polymer chain segments.^[^ ^32^ ^]^ The evaporation of ethanol reduced the space occupied by the solvent phase and self‐assembly of the polymer chains formed a denser oriented network. Then, the PVA/glycerol organogel underwent wet‐annealing in hot‐stretching which further promoted the orientation and crystallization of the macromolecular chains, yielding aligned nanofibrils. Finally, the PVA/glycerol organogels were transformed to hydrogels by secondary solvent exchange with water, and the anisotropic structure was maintained in the hydrogel caused by the strong inter‐macromolecular interactions. Figure 1c,d compares the structures of natural tendons and AH‐200, which indicate that both have highly similar structures comprising oriented fiber bundles. At the nanoscale, natural tendon has directionally aligned helical collagen molecules, and AH‐200 has aligned nanofibrils made up of oriented macromolecular chains. Also, the cross sections of fibers in AH reveal a dense microporous structure with pore sizes of a few tens of nanometers, resembling the endomysia of tendon (Figure S3, Supporting Information). It is noted that tendons are macroscopically opaque, while AHs have transparencies greater than 85% (Figure S4, Supporting Information). In addition, AH‐200 is strong to hold an 18 kg dumbbell without failure (Figure S5, Supporting Information) and can withstand pinpricks (Figure 1d). In the following sections, the mechanical properties of AHs including their fatigue resistance are shown and discussed. Tendons have tensile strengths in the range 13 to 124 MPa and water content ≈60%.^[^ ^33^, ^34^ ^]^ Since the AHs are similar to natural tissues, they are excellent candidates for tendon repair.

### 2.2 Mechanical Properties of AHs

**Figure 2:**
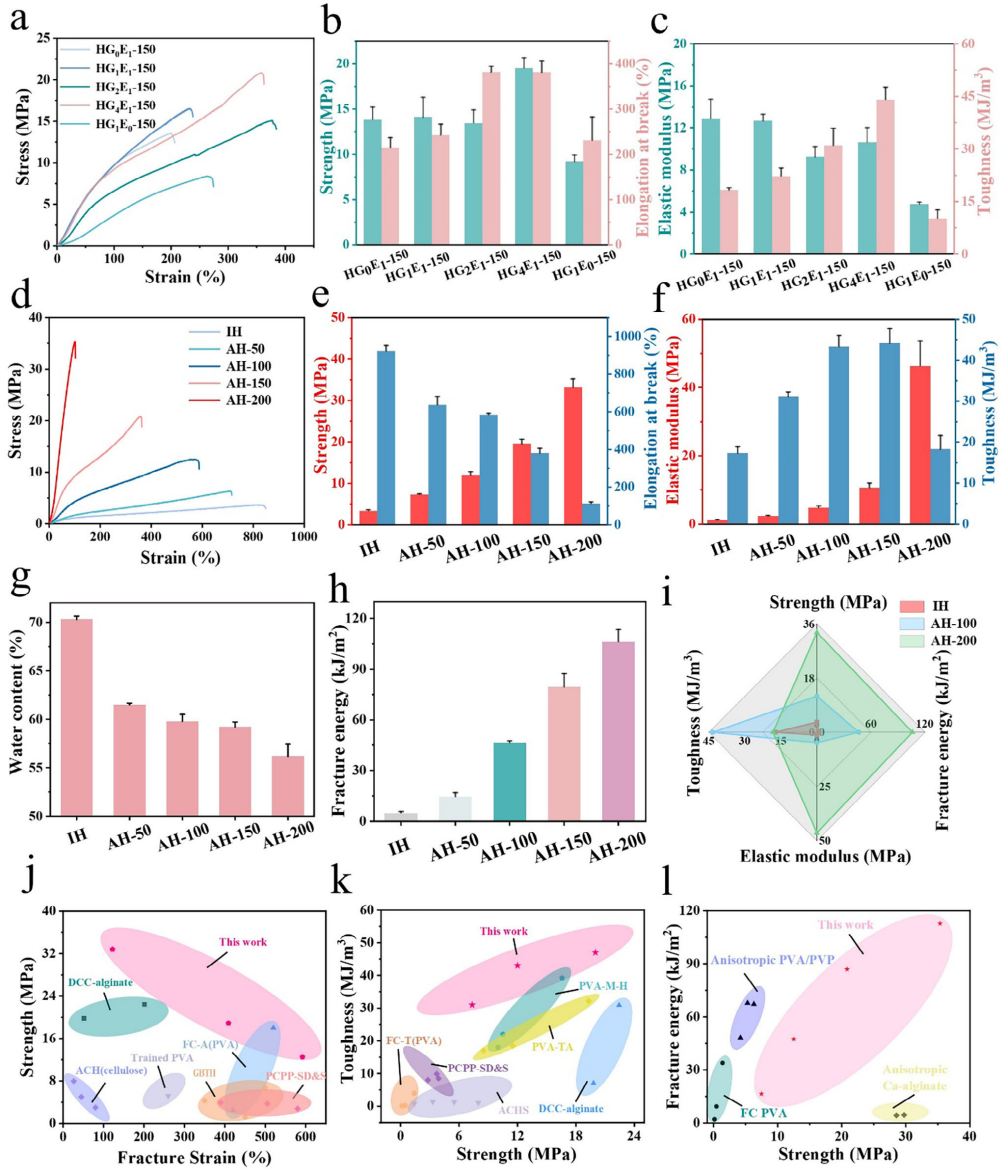
Mechanical properties of AHs. a) Stress‐strain curves, and summary of b) tensile strength and elongation at break, and c) elastic modulus and toughness of AHs prepared by different glycerol/ethanol ratios. d) Stress‐strain curves, and summary of e) tensile strength and elongation at break, and f) elastic modulus and toughness of AHs prepared with different pre‐stretching ratios (PSRs). g) Water content of hydrogels prepared by different PSRs. h) Fracture energy of AHs with different PSRs. i) Summary of different mechanical properties of isotropic PVA hydrogel (IH), AH‐100 and AH‐200. Comparison of j) tensile strength versus fracture strain, k) toughness versus tensile strength, and l) fracture energy versus tensile strength of AHs and other anisotropic hydrogels.

As mentioned in Section 2.1, the volatile poor solvent evaporation during hot‐stretching significantly promoted macromolecular chain alignment and thus enhanced the mechanical properties. First, we explored the effect of glycerol/ethanol ratio on mechanical properties of isotropic hydrogels without pre‐stretching (Figure S6, Supporting Information). HG_1_E_0_ showed tensile strength and elongation at break of 4.32 MPa and 1018.72%, respectively, and toughness of 21.82 MJ m^−3^. But adding ethanol generally decreased tensile strength, toughness and elongation at break of the hydrogels even though the elastic modulus was increased. HG_4_E_1_ also exhibited the highest water content of 71% (Figure S7, Supporting Information) of all hydrogels. However, ethanol was indispensable in improving the mechanical properties of AHs, and if it was absent for preparation of AHs, the obtained hydrogels would show weak mechanical performances. Because during the hot stretching process, ethanol volatilized, which increased the stress in the stretching direction, promoted the orientation and crystallization of macromolecular chains, and facilitated the formation of well – aligned nanofibrils. **Figure** **2**a displays typical tensile stress‐strain curves of different hydrogels, and it is found that HG_4_E_1_‐150 and HG_1_E_0_‐150 have the best and worst mechanical properties. Figure 2b summarizes detailed mechanical properties of the hydrogels. HG_1_E_0_‐150, HG_1_E_1_‐150, HG_2_E_1_‐150, HG_4_E_1_‐150, and HG_0_E_1_‐150 showed tensile strengths of 9.21 ± 0.72 MPa, 14.12 ± 2.16 MPa, 13.46 ± 1.45 MPa, 19.54 ± 1.12 MPa and 13.83 ± 1.39 MPa with matching elongations at break of 230.87 ± 51.55%, 242.37 ± 23.96%, 381.42 ± 12.89%, 380.82 ± 24.77% and 214.79 ± 22.02%. Also, the toughness of HG_4_E_1_‐150 is as high as 44.1 MJ m^−3^ (Figure 2c), much higher than 10.24 MJ m^−3^ for HG_1_E_0_‐150 and 18.33 MJ m^−3^ for HG_0_E_1_‐150. When ethanol is absent, although hot stretching alone still leads to some improvement over isotropic hydrogels, the enhancement in mechanical properties is significantly reduced. This limitation is attributed to the absence of ethanol‐mediated intermolecular interactions, and the diminished stress‐promoting effect during stretching. When only glycerol is used as the solvent, its ability to facilitate ordered molecular chain alignment and cross‐linking is markedly weaker compared to mixed solvent of glycerol and ethanol. Besides, in the absence of pre‐stretching, PVA chains largely remain in a disordered state. Although variations in ethanol content may influence intermolecular interactions, these non‐pre‐stretched hydrogels lack the hierarchical energy‐dissipating structures observed under pre‐stretched conditions. These results confirm the synergy of volatile poor solvent evaporation, pre‐stretching and wet‐annealing enhancement of mechanical properties. Figure 2d–f shows the mechanical properties of hydrogels prepared with different PSR. Clearly, the hydrogel tensile strength increases with PSR, but the elongation at break shows a reverse trend with a continuous decrease. AH‐200 exhibited the highest tensile strength of 35.34 MPa, which is almost 10 times that of isotropic PVA hydrogel (IH) (3. 65 MPa), while its elongation at break was only 122%, much lower than 942.69% for IH. In addition, with the increase of PSR from 50% to 200%, the Young's modulus was dramatically increased from 2.4 to 46.37 MPa. Despite its highest tensile strength and elastic modulus, AH‐200 possessed the smallest toughness of ≈18.27 MJ m^−3^ of all AHs, which is only comparable to that of IH. The sacrifice of the toughness was caused by the highly oriented structure that severely limited the stretchability of the macromolecule chains. Conversely, AH‐150 showed the largest toughness of 44.1 MJ m^−3^ and satisfactory strength of 20.8 MPa, hence achieving the balance between stiffness and toughness.

It is well‐known that there is usually a conflict for hydrogels between good mechanical properties and high water content. Usually, the mechanical properties decrease with increase of water content. In our work, the water content of IH was ≈68%. For AHs, it decreased with increasing PSR, and was ≈60% and ≈57% for AH‐150 and AH‐200 (Figure 2g), respectively. The water content could be tuned by varying the PVA concentration. When this was decreased to 10 wt.%, the obtained anisotropic hydrogel with PSR of 200% possessed a water content of ≈66%, with a tensile strength of 12.6 MPa (Figure S8, Supporting Information). These results confirm that the poor solvent evaporation and wet‐annealing assisted pre‐stretching strategy has unique advantages for fabrication of anisotropic, strong and tough hydrogels with widely tunable mechanical properties and water content.

The mechanical properties and water content of hydrogels were improved by the synergistic effect of solvent volatilization, wet annealing and pre‐stretching. IH is known to exhibit isotropic mechanical properties, while large dissimilar mechanical properties were observed for AHs in the parallel and transverse directions. As shown from the stress‐strain curve in Figure S9 (Supporting Information), AH‐100 displayed a much higher tensile strength in the parallel direction (12 MPa) than that in the transverse direction (2.97 MPa), and the fracture strain decreased from 838% to 584%. It is noted that the mechanical properties of AH‐100 even in the transverse direction were comparable to those of IH. We conducted further investigations into the dynamic viscoelastic characteristics of the hydrogels. The storage modulus (*G'*) values of IH and AH‐100 were larger than the loss modulus (*G“”*) values, exhibiting solid‐like and elasticity‐dominated properties (Figure S10a, Supporting Information). *G'* and *G“”* in the frequency range 0.1 to 100 rad s^−1^ of AH‐100 were higher than IH due to the strong intermolecular interaction (Figure S10b, Supporting Information). More importantly, AHs showed remarkable resistance to crack growth. Figure S11 (Supporting Information) exhibits the force‐displacement curves of notched and unnotched PVA hydrogels. The fracture energy increased hugely with PSR, reaching to an extraordinarily high value of 112.73 kJ m^−2^ for AH‐200, which is ≈20 times that of IH (5.8 kJ m^−2^) (Figure 2h). Figure 2i compares the mechanical properties of IH with those of AH‐100 and AH‐200. Clearly, the anisotropic hydrogels have superior mechanical properties than IH, and AH‐200 illustrated advantages in tensile strength, elastic modulus and fracture energy but showed a disadvantage in toughness. Figure 2j–l summarizes the state of the art for anisotropic hydrogels reported in the literature.^[^ ^16^, ^18^, ^22^, ^25^, ^26^, ^27^, ^35^, ^36^, ^37^, ^38^ ^]^ Undoubtedly, AHs demonstrated superior overall mechanical properties relative to the other reported anisotropic hydrogels (containing no ions) by pre‐stretching or directional freezing. In particular, AH‐200 displayed markedly greater strength, toughness, and fracture energy.

### 2.3 Structures of AHs

To clarify the origin of the excellent mechanical properties, we performed a series of structure characterizations of IH and AHs. IH showed an interconnective porous structure with pore size of ≈256 nm (Figure S12, Supporting Information), while directionally aligned fiber structure is present for AHs, as shown from the SEM image (Figure 1d), and it was also verified by AFM and optical microscope images (Figure S13, Supporting Information). In addition, the transverse section of AH‐200 showed a homogeneous porous yet dense structure (Figure S3, Supporting Information), but the pore size was greatly reduced compared to that of IH, which was closely related to the anisotropy and water content.

**Figure 3:**
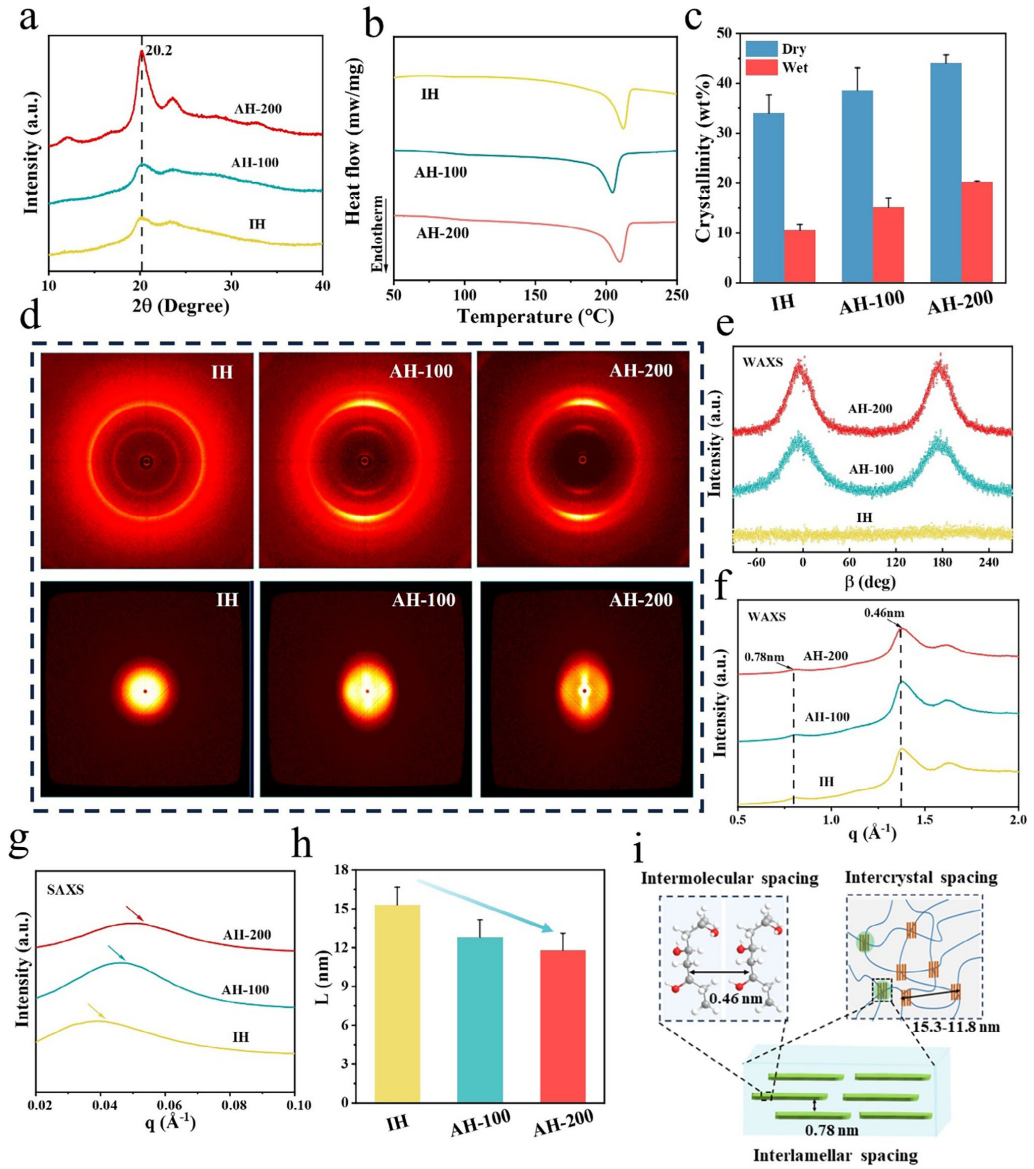
Structural characterization of IH, AH‐100, and AH‐200. a) XRD patterns. b) DSC thermographs, and c) corresponding calculated crystallinities. d) 2D WAXS and 2D SAXS patterns. e) Correlation of the azimuthally integrated intensity distribution of 2D WAXS patterns. f) Scattering intensity versus scattering vector *q*. g) Corrected scattering intensity *Iq^2^* versus vector *q*. h) Calculated average distance between crystalline regions. i) Schematic illustration of intermolecular spacing, interlamellar spacing and intercrystal spacing of AHs.

The FTIR spectra (Figure S14, Supporting Information) showed that the characteristic stretching vibration peak of the hydroxyl groups is located at ≈3200–3400 cm^−1^, and is shifted to the region of low‐wavenumber with increasing PSR, from 3303 cm^−1^ for IH to 3291 and 3286 cm^−1^ for AH‐100 and AH‐200, respectively. Compared to IH, the intermolecular hydrogen bonding interaction was enhanced in AHs, which was consistent with the mechanical properties of the hydrogels. To investigate the impact of pre‐stretching on crystallinity, the crystallization behavior was first characterized by X‐ray diffraction (XRD) (**Figure** **3**a). When the PSR was increased, the crystallization peak of PVA at 2θ = 20.2° became increasingly intense, and AH‐200 exhibited a much sharper crystallization peak than the other two hydrogels. To determine the crystallinity quantitatively, the crystallization behavior of the hydrogels in the dry state was investigated by DSC test, and the crystallinity in the swollen state was accordingly calculated. It was found that the crystallinity of IH was lower than that of AHs, and AH‐200 showed the highest crystallinity of 44% and 22% in the dry and swollen states, respectively (Figure 3b,c). These results indicate that more crystalline domains were formed with a larger PSR, and the cross‐linking density of the macromolecular chains in the hydrogels and thus the tensile strength and elastic modulus were increased. To further quantify the evolution of the crystal morphology, wide‐angle and small‐angle X‐ray scattering (WAXS) and (SAXS) tests were conducted. As can be seen from the WAXS patterns, IH showed uniform diffraction patterns at all azimuthal angles of the scattering plane, indicating a distinct isotropic structure (Figure 3d). By contrast, AH‐100 and AH‐200 have strong angular dependence of their equatorial arcs, exhibiting an obvious anisotropic structure. The azimuthal integral angle distribution curves further confirmed the oriented structure of AHs, and the azimuthal angle became increasingly sharp as PSR increased (Figure 3e). The degree of orientation of IH was 0, while those of AH‐100 and AH‐200 were 0.72 and 0.78, respectively. This confirms that the poor solvent evaporation‐assisted hot‐stretching process is highly effective in aligning the macromolecular chains (Figure S15, Supporting Information). As shown in Figure 3f,i, the strongest peak in the WAXS 1D patterns was present at 0.46 nm (the average distance between adjacent PVA chain segments of the nanocrystalline region), and the weakest peak appeared at 0.78 nm (the average layer spacing in the nanocrystalline domains). From the 2D SAXS patterns, it can be seen that the scattering intensities of IH in all directions are almost the same. Furthermore, the scattering patterns of AH‐100 and AH‐200 gave an elliptical shape, and the ellipticity was more obvious for AH‐200 (Figure 3d). AH‐50 and AH‐150 also display this characteristic feature (Figure S16, Supporting Information). The 2D SAXS azimuthal integration curves also showed that the azimuthal intensity was positively correlated with PSR (Figure S17, Supporting Information), and the calculated orientations were similar to those determined from the WAXS mode (Figure S18, Supporting Information). In addition, the peak shifted to the high scattering vector by increasing PSR, leading to continuous decrease of the distance between adjacent crystalline domains (Figure 3g,h). The average distance between neighboring crystalline domains of IH, AH‐100, and AH‐200 were calculated to be 15.3, 12.8, and 11.8 nm, respectively. From the XRD results, the average size of the crystals increased from 6.59 nm for IH to 6.64 nm and 7.0 nm for AH‐100 and AH‐200, respectively (Figure S19, Supporting Information). The detailed size information about the crystalline structure of AH‐100 and AH‐200 was summarized in Figure 3i.

Here, the poor solvent evaporation enhances the tensile stress along the stretching direction, and the ensuing hot‐stretching reduces the resistance of macromolecule movement, facilitating adjustment of macromolecular chain conformation and hastening the orientation of macromolecules. In addition, increased PSR promotes regular arrangement of macromolecular chains, and produces more crystalline domains with increased crystal size but reduced inter‐domain distance. Hence, inter‐macromolecular interactions, such as hydrogen bonding, are strengthened. Meantime, from a macroscopic perspective, more crystalline domains and straightened macromolecular chains improve the hydrogel anisotropy and densification. The increased crystallinity provides more physical cross‐linking points for effective stress transfer. In addition, aligned macromolecular chains with enhanced inter‐chain interactions can improve the load‐bearing capabilities of the hydrogels.

### 2.4 Anti‐Fatigue Performance of AHs

**Figure 4:**
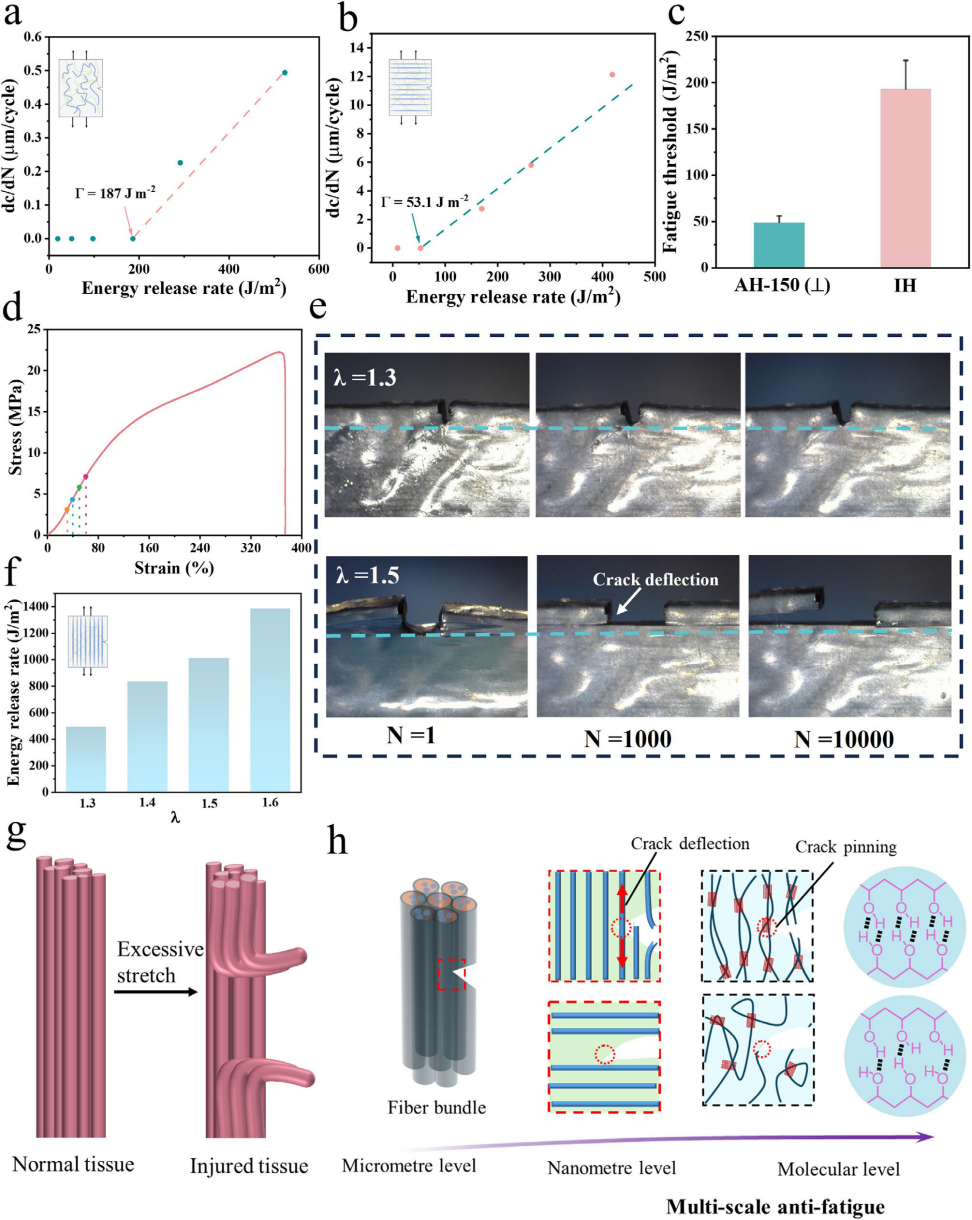
Fatigue resistance of AHs. Crack propagation per cycle, dc/dN versus applied energy release rate *G* for a) IH and b) AH‐150 (notch parallel to aligned fiber direction), and c) summary of corresponding fatigue thresholds. d) Stress‐strain curve of AH‐150. e) Micrographs of the crack before and after 1000 and 10 000 cycles for AH‐150. Scale bar: 500 µm. f) Energy release rate (*G*) for AH‐150 in fibril alignment direction. Schematic for g) simplified tearing model of soft tissues, and h) anti‐crack propagation mechanism of AHs at different length scale.

The performance of hydrogels under static loading is different from that under cyclic loading, and high strength and toughness do not mean excellent fatigue resistance.^[^ ^39^, ^40^, ^41^, ^42^ ^]^ Particularly, many hydrogels show weak crack propagation resistance, severely limiting their applications as load‐bearing materials. 1000 cyclic tensile tests were performed, and all hydrogels exhibited evident mechanical hysteresis in the first cycle (Figure S20, Supporting Information), known as the Mullins effect.^[^ ^43^ ^]^ As PSR increased, the hysteresis loop became more obvious, and the dissipated energy rose from 29.5 kJ m^−3^ for IH to 453 kJ m^−3^ and 843 kJ m^−3^ for AH‐150 and AH‐200, respectively (Figure S21, Supporting Information). As discussed, higher PSR gives rise to enhanced hydrogen bonding and crystallinity, and more energy is thus required to break the hydrogen bonds and orientate the crystalline domains during stretching. After 50 cycles, the loading and unloading curves almost overlap without any hysteresis loops, i.e., the dissipated energy was close to zero, and the maximum stress at 1^st^ cycle for AH‐150 and AH‐200 were 10.2 MPa and 18.6 MPa, respectively. Fatigue threshold, below which there was no crack growth due to cyclic loading, was determined for the hydrogels. IH showed a fatigue threshold of 187 J m^−2^ (**Figure** **4**a), and the crack started to extend with λ (λ = 1+ε, ε: tensile strain) equal to 1.3. When the initial crack of AH‐150 was parallel to the aligned nanofibrils, it propagated rapidly at λ = 1.2 and it showed the fatigue threshold of 53.1 J m^−2^ (Figure 4b,c). As shown in Figure 4d–f, when the initial crack was perpendicular (or transverse) to the direction of aligned nanofibrils, the crack did not spread under 10000 loading cycles when λ was smaller than 1.3 equal to an energy release rate of 495.5 J m^−2^. When λ was increased to 1.4, 1.5, and 1.6, crack deflection and branching along the aligned fibrils direction was seen at 1000 and 10000 load cycles with respective energy release rates of 834.9,1011.6, and 1386.3 J m^−2^. Under cyclic loading, and owing to the anisotropy, the cracks could not propagate along the initial perpendicular direction but deflected/branched as described earlier. This special flaw‐insensitivity feature effectively protects the notched hydrogel without catastrophic failure across the whole cross section. Hence, AHs deflected cracks in the direction of loading and deflection/branching results in peeling off the sample, hence leaving the remaining section intact. Biological tissues, such as muscles and tendons, are assembled from ordered protein fibers, and these fibrillar soft tissues can break under excessive tension and tear along the weak interfaces between fibers to prevent further damage to the entire tissue (Figure 4g).^[^ ^44^, ^45^ ^]^ Here, AH‐150 also displays similar anti‐crack growth and self‐protection behavior. The AHs in this study have unique hierarchical structure, viz., the densified polymeric network consists of fiber bundles with embedded nanofibrils which comprise aligned macromolecule chains, crystalline domains and intermolecular hydrogen bonds. Therefore, multi‐level reinforcement, schematically shown in Figure 4h, is proposed to reveal the unique crack resistance mechanisms of AHs. The exceptional defect tolerance of the PVA hydrogel arises from its hierarchically orchestrated energy dissipation architecture, which operates across three synergistic scales to suppress crack propagation. At the micrometer level, when the crack is perpendicular to the aligned fiber bundles and the specimen is being stretched vertically, it continues to grow in its original direction across the fibers and are then pinned by the high‐strength fiber bundles, and finally deflects along the weaker interfaces. In the parallel direction, the high‐strength fiber bundles impede crack propagation along the initial path. The crack tip region exhibits a self‐training effect that effectively mitigates local stress concentration and decelerates the crack propagation rate.^[^ ^46^ ^]^ The aligned fibers induce anisotropic stress redistribution, which promotes crack deflection and consequently reduces the stress intensity at the crack tip. At the nanoscale, the nanofibers that are directionally arranged within the fiber bundles serve as the secondary reinforcing phase. The nanofibrils can bridge the crack, be broken and pulled out, hence dissipating substantial energy. At the molecular scale, the crystalline domains serve as rigid and highly functional cross‐linkers and can stop or pin the crack, increasing the crack growth resistance. Moreover, the numerous hydrogen bonds are also responsible for further energy dissipation. During stretching, the macromolecule chains disentangle and slip, damaging hydrogen bonds. Dynamic reconstruction of reversible hydrogen bonding dissipates a large amount of energy. This fracture behavior is analogous to that observed in a silicone elastomer where cracks undergo stable propagation with directional deflection perpendicular to the initial notch orientation.^[^ ^47^ ^]^ The lateral crack length exhibits gradual extension with increasing load, while crack arrest occurs immediately upon load removal. Summarizing, these anisotropic hydrogels with cracks transverse to the aligned fibers are only partially damaged with a self‐protection function.^[^ ^48^ ^]^ However, it should be realized that when the crack is parallel to the direction of the aligned fiber bundles under the same vertical loading, it can easily propagate in the weak interstitial regions consisting of amorphous macromolecular chains between fiber bundles, causing rapid failure of the hydrogels.

### 2.5 Anti‐Swelling Behavior and Biocompatibility

**Figure 5:**
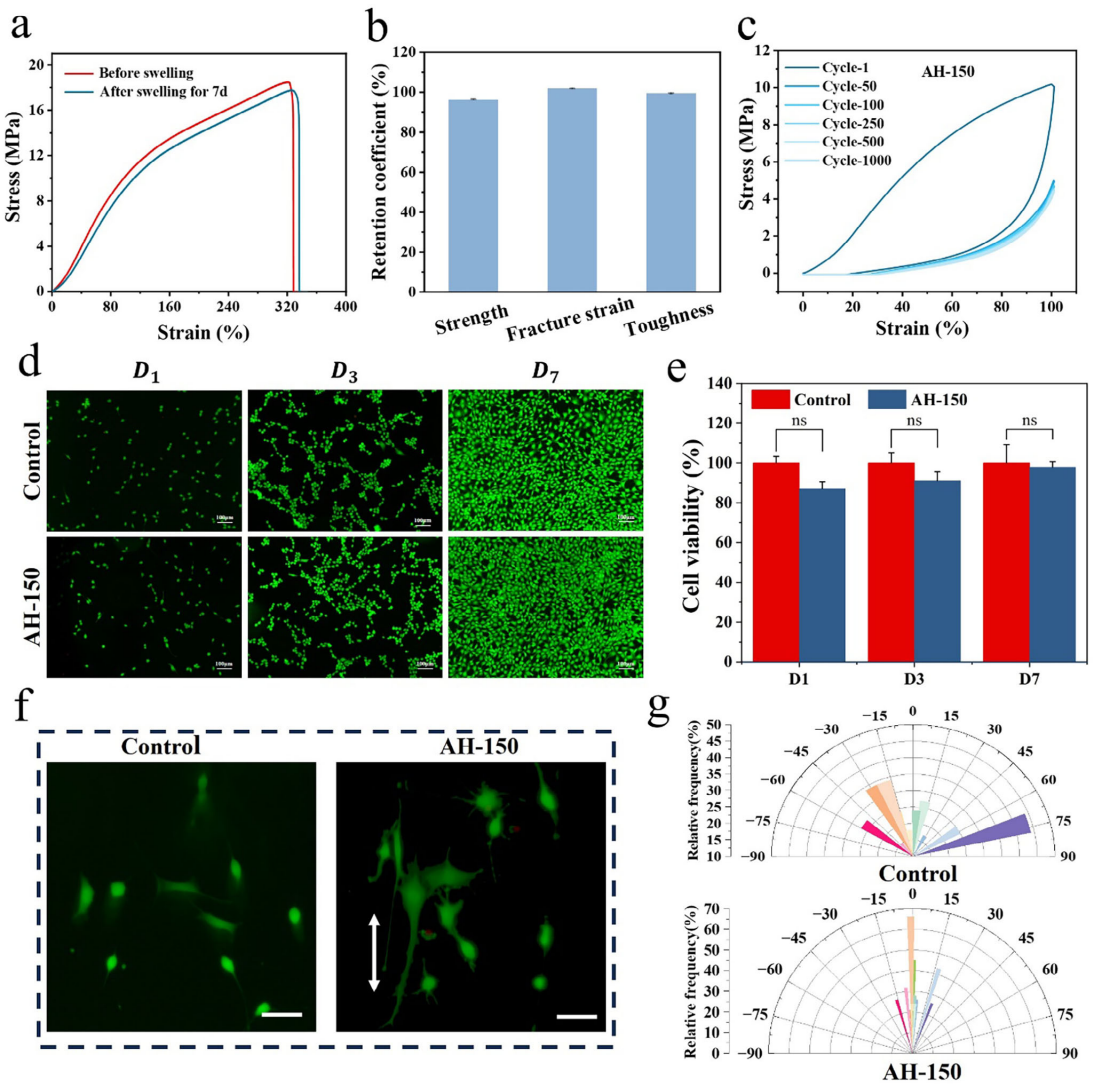
a) Typical tensile stress‐strain curves of AH‐150 before and after swelling in water for 7 days, and b) retention coefficient of mechanical properties c) Stress versus strain curves over 1000 cyclic tensile tests of AH‐150 in a water bath. d) LIVE/DEAD staining images of NIH/3T3 cells upon contacting directly cell culture plate (control) and AH‐150 for 1, 3, and 7 d. e) Proliferation abilities. f) Fluorescence images of NlH‐3T3 fibroblasts cultured on different substrates and g) distribution of orientation angles for the Control group and AH‐150 (*n* ≥ 20), scale: 100 µm.

Most high‐strength hydrogels typically swell in water and reduce their long‐term durability, hence limiting their use in living organisms. To assess the swelling resistance of AH‐150, it was immersed in water, physiological saline, and PBS solution for 3 d and 7 d, respectively. After 7 d, the swelling rate in water, physiological saline and PBS solution was less than 5% (Figure S22, Supporting information). AHs were prepared through solvent exchange of glycerol with water, and the macromolecular network had almost reached an equilibrium state in water, thus the obtained hydrogels hardly swell when they are used in underwater environment. Further, the excellent anti‐swelling performance originates from the densified crystallized domains, which yield many cross‐linking points, preventing macromolecule swelling. **Figure** **5**a shows the stress‐strain curve of AH‐150 before and after swelling in water for 7 d. The tensile strength and fracture strain of decrease slightly after swelling with the retention coefficient of 96% and 99%, respectively. The fracture strain is also almost unchanged (Figure 5b). The excellent anti‐swelling properties of AH‐150 ensure its size stability and durable mechanical properties in living organisms. Figure 5c shows the cyclic loading‐unloading curves of AH‐150 under 100% strain in water. After 50 cycles, the tensile stress tends to be stabilized, indicating that AH‐150 has long‐term durability in living organisms, and can be so used. Cytotoxicity should be carefully considered before hydrogels are used for tissue engineering. Cell Counting Kit‐8 tests on mouse embryonic fibroblasts (NIH‐3T3) and LIVE/DEAD staining were used to evaluate the cytocompatibility of the hydrogel. As shown in Figure 5d, cells grown on an empty tissue culture plate were used as the Control group. The cells were stained using a live/dead staining method in which live cells were stained green with calcein‐AM and dead cells were stained red with propidium iodide. Only a small number of dead cells were detected on the surface of AH‐150, which is similar to the phenomenon seen in the Control group. As shown in Figure 5e, the survival rate of AH‐150 and NIH‐3T3 co‐cultured for 1, 3, and 7 d was larger than 85%. Particularly, the survival rate on the 7^th^ day was up to 97.92% ± 2.71%, and showed no significant difference with the Control group, indicating that AH‐150 had good biocompatibility. Cell morphology can be regulated by various structural factors of the extracellular matrix, e.g., stiffness anisotropy and surface topography.^[^ ^49^, ^50^ ^]^ Since AH‐150 comprises aligned fibrils as well as anisotropic structure and properties, these anisotropic characteristics will also influence cell growth and morphology (Figure 5f,g). After incubation for 24 h, the cells in the Control group (blank medium plate) were randomly adhered to the wall; whereas AH‐150 showed significantly directed cell growth with the cell orientation concentrated in the range ‐16° to 21°. It is seen that under the induction of the anisotropic structure on the surface of AH‐150, the cell pseudopodia extended directionally. Hence, the aligned structure can control the morphology and function of the cells. Aligned fibrils enhance tendon gene expression, and also promote the formation of parallel‐arranged collagen fibers and tendon‐like tissues.^[^ ^31^ ^]^

### 2.6 Reconstruction of Tendons After Injury

**Figure 6:**
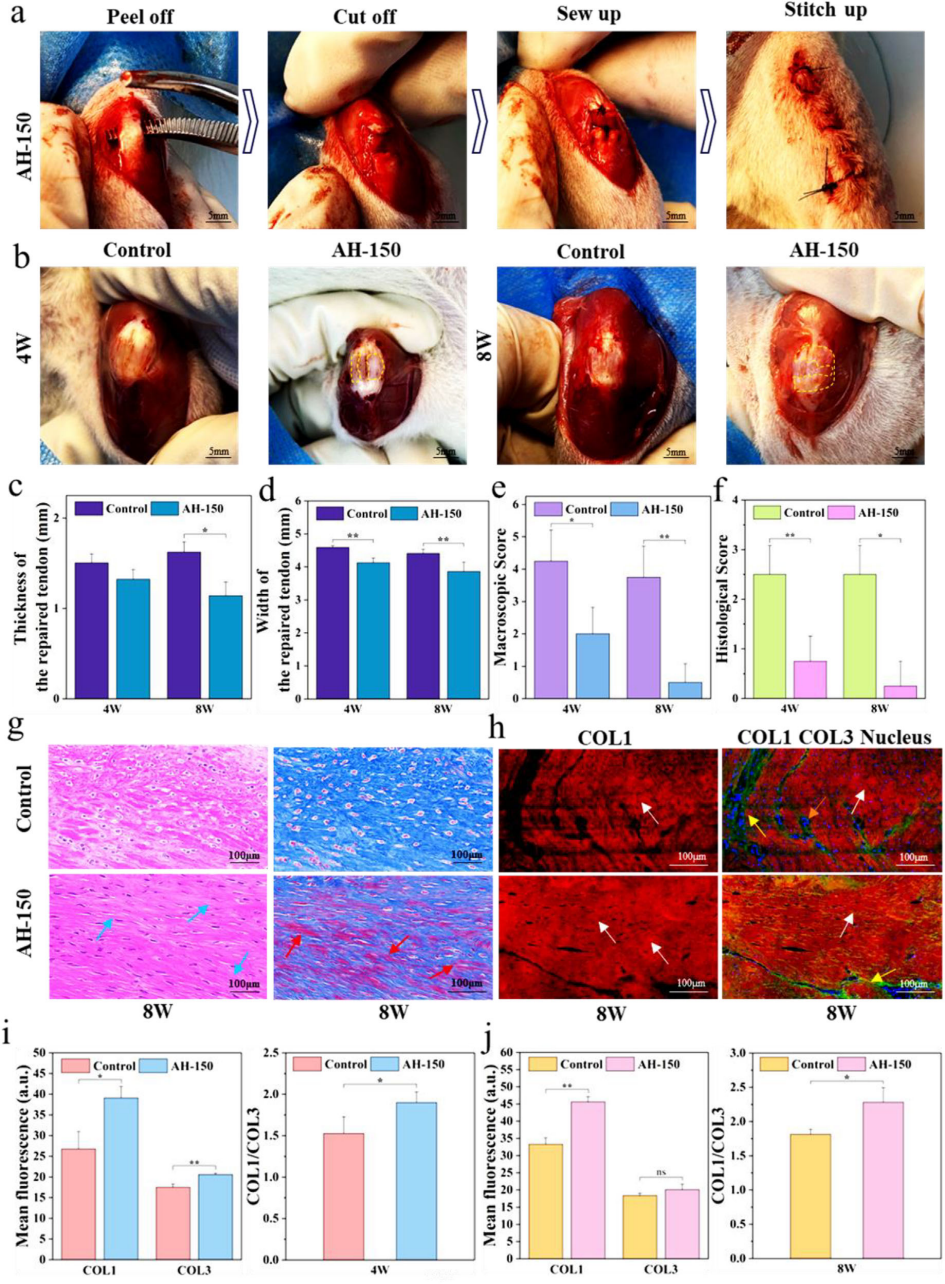
Tendon repair performance of AH‐150. a) Surgical procedures: I) peel off tendon, II) cut off mid‐portion of the tendon, III) suture the hydrogel to both sections of the severed tendon, and IV) suture the wound. Scale bar: 5 mm. b) Typical macroscopic appearance of repaired tendons. Scale bar: 5 mm. c,d) Thickness and width of the repaired tendon, *n* = 4. e) Macroscopic score of the two groups, n = 6. f) Histological score based on H&E and Masson staining, *n* = 6. g) H&E and Masson staining of tendon tissues at the 8^th^ week. Blue arrows indicate densely aligned fibers and red arrows indicate muscle fibers. Scale bar: 100 µm. h) Tendon immunofluorescence staining images of COL1 (red), COL3 (green), and nucleus (blue) at 8 weeks and results statistics, white arrows indicate COL1, yellow arrows indicate densely COL3, orange arrow indicate nucleus. Scale bar: 100 µm, n = 6. i) Statistics of tendon immunofluorescence staining of COL1, COL3 and COL1/COL3 at 4 weeks. j) Statistics of tendon immunofluorescence staining of COL1, COL3 and COL1/COL3 at 8 weeks. (*) *P* < 0.05, (**) *P* < 0.01, and (***) *P* < 0.001.

AH‐150 has microstructures and properties similar to those of natural tendons. The mechanical properties of hydrogels (strength and fatigue resistance) influence tendon repair efficiency through multiple mechanisms, including early mechanical function substitution, promotion of extracellular matrix (ECM) synthesis to enhance tendon regeneration,^[^ ^51^ ^]^ prevention of secondary injury,^[^ ^52^ ^]^ and reduction of inflammatory adhesion.^[^ ^53^ ^]^ The favorable mechanical adaptability of the AH‐150 material in this study provides a solid basis for its application in tendon repair. We replaced the injured tendons of rats with AH‐150 to evaluate the repair performance of rat tendons, capitalizing on the bio‐compatibility and excellent mechanical properties of the hydrogel. As shown in **Figure** **6**a and Figure S23 (Supporting Information), full‐thickness tendon defects were produced by stripping and cutting the bilateral patellar tendons of female SD rats (Sprague‐Dawley rats, 5–6 weeks old). AH‐150 was sutured to the injured tendon to replace tendon tissue. As a Control group, the injured tendon was sutured directly (Figure 6a). At 4 and 8 weeks, the damaged area of the tendon in AH‐150 was well filled with regenerated tissue and its surface was smooth. The surface of the regenerated tendon also has a milky‐white tissue appearance similar to that of the natural tendon (Figure 6b). At 8 weeks, the milky‐white tissue area in the regenerated tendon in the AH‐150 group was significantly larger. Figure 6c,d shows that at 4 and 8 weeks, the thickness and width of regenerated tendons in the AH‐150 group were reduced. These results suggest that tendons in the AH‐150 group inhibited scar formation during the repair process. Besides, anatomical observation of the rat patellar tendon site at 8 weeks post‐surgery (Figure S24, Supporting information) revealed that the volume of the AH‐150 material at the implantation site decreased significantly, with a degradation rate of 36.49% ± 8.09%, confirming its in vivo degradability. As displayed in Figure 6e, tendon repair in the AH‐150 group had a much lower macroscopic score than the Control group measured by multiple factors such as inflammation, tendon adhesion, defect area filling, shape and color.^[^ ^54^ ^]^ The regeneration of tendon after implantation of AH‐150 was evaluated by observation of tissue section images of the regenerated tendon at different times. The early stage of tendon repair is the proliferative stage characterized mainly by an increase in the number of tenoblasts with disordered arrangement and collagen synthesis.^[^ ^12^ ^]^ The late stage of tendon repair is the remodeling stage, which is characterized by a more orderly arrangement of collagen fibers and an increase in the tensile strength of the regenerated tendon. H&E and Masson trichrome staining were applied to reveal the morphological organization and collagen fiber distribution within the repaired tissue. As shown in Figure 6g (8 weeks) and Figure S25 (Supporting Information) (4 weeks), the AH‐150 group had a higher number of mature tenoblasts and tenocytes, with a tighter distribution in H&E staining. In addition, the morphology and distribution of the cells were predominantly subcircular and regionally arranged at 4 weeks, and gave a spindle‐shaped morphology and parallelly arranged at 8 weeks. Masson's trichrome staining further revealed the reorganization of collagenous tissue during tendon healing. Upon analyzing the Masson stained images at 8 weeks, it is evident that the regenerated tendons of AH‐150 group exhibited an abundance of newborn collagen fibrils, accompanied by a high degree of directional organization. Moreover, the AH‐150 group displayed a higher formation of myofibers which possessed contractile capabilities, thus enhancing the tensile strength of the tendon and facilitating blood perfusion to the tendon tissues. Figure 6f shows that the histologic score of the AH‐150 group was significantly lower than that of the Control group. The results indicate that AH‐150 is beneficial for remodeling and regeneration of tendon tissues. Also, implanted AH‐150, acting as a bridge to promote the production of new collagen fibers, facilitates directed cell growth and assists proliferation of tendon cells during early stages of healing. This provides favorable conditions for subsequent tendon tissue regeneration. Collagen regeneration in different groups was based on the observation of immunofluorescence stained images of the regenerated tendons at 4 and 8 weeks. Collagen type I (COL1) is the major structural component of the tendon.^[^ ^55^ ^]^ Also, collagen type III (COL3) plays a pivotal role in the architectural development of nascent tissue during the initial phases of tendon repair and regeneration. During the remodeling phase, mechanical loading is required for the tendon to change collagen type III (COL3) to collagen type I (COL1).^[^ ^56^, ^57^ ^]^ The expression level of COL1/COL3 was critical for the physiological healing of the tendon during repair.^[^ ^58^ ^]^ Figure 6h (8 weeks) and Figure S26 (Supporting Information) (4 weeks) show that the staining area of COL1 was larger, and the fluorescence intensity was stronger in the AH‐150 group. Combined with the quantitative data in Figure 6i,j, it is clear that the regeneration of COL is much faster in the AH‐150 group, which indicates that the implantation of AH‐150 promotes collagen regeneration. As shown in Figure S27 (Supporting Information), we systematically compared AH‐150 with reported hydrogel systems at 4 and 8‐week post‐implantation.^[^ ^16^, ^59^, ^60^ ^]^ Quantitative analyses revealed that the AH‐150 group exhibited significantly higher COL1 expression than other hydrogels at 4 weeks (early repair phase), indicating enhanced early‐stage healing. By 8 weeks (maturation phase), the COL1/COL3 ratio in AH‐150‐treated tendons approached the typical levels observed in healthy tendons.^[^ ^61^ ^]^ These results indicate that AH‐150 effectively facilitates early tendon repair and late‐stage remodeling, providing a more promising option for treating tendon injuries.

## 3 Conclusion

In this work, we reported a strategy of “poor solvent evaporation assisted hot‐stretching” for the development of anisotropic, super‐strong and flaw‐insensitive hydrogels. AHs show excellent mechanical properties with tensile strength and fracture energy as high as 33.14 ± 2.05 MPa and 106.18 ± 7.2 kJ m^−2^, respectively. Besides, AHs exhibit outstanding fatigue resistance, and cracks can only spread or deflect along the fiber orientation direction but cannot propagate transverse to the fibrous structure, displaying an intrinsic self‐protection function akin to the tearing of biological soft tissues. Furthermore, AHs with superior biocompatibility and swelling resistance can also be used for repair of rat tendons, and implanted AHs can promote collagen regeneration to facilitate tendon repair.

## 4 Experimental Section

### Materials

Polyvinyl alcohol (PVA‐1799, 98–99% hydrolyzed, Aladdin), glutaraldehyde (50 volume%, Aladdin), dimethyl sulfoxide (DMSO, Aladdin), ethanol absolute (AR), fluorescein sodium salt (Aladdin) and glutaraldehyde (Aladdin) were used without further purification. Fetal bovine serum (FBS) and Dulbecco's Modified Eagle Medium (DMEM) were supplied by American Gibco (Gaithersburg, MD, USA). Penicillin/ streptomycin (PS), calcein‐acetoxy methylester (AM), and propidium iodide (PI) were bought from Thermo Fisher Scientific Inc. (Waltham, MA, USA). Cell Counting Kit‐8 (CCK‐8) was purchased from Dojindo Laboratories (Kumamoto, Japan). Paraformaldehyde (PFA; 4.0%, *W/V*) was obtained from Aladdin Biochemical Technology Co., Ltd. (Shanghai, P. R. China). All aqueous solutions were prepared using ultra‐pure deionized water.

### Preparation of PVA Solution

10 wt.% and 15 wt.% PVA solutions were prepared by mixing PVA powders with DMSO under stirring and heating (90 °C, 3 h).

### Fabrication of Isotropic PVA Hydrogels

The PVA/DMSO solution was poured into a PTFE mold and immersed in glycerol/ethanol mixture for 48 h, and the weight ratio between glycerol and ethanol was set to 1:0, 4:1, 2:1, 1:1, and 0:1. The PVA/glycerol/ethanol organogels underwent wet‐annealing for 0.5 h at a temperature of 120 °C. Finally, it was subsequently immersed in deionized water. The organogel was placed in a beaker containing 1000 mL of deionized water for 48 h of solvent exchange, and the deionized water was changed at least three times a day to ensure that the residues were replaced by water.

### Fabrication of Anisotropic PVA Hydrogels

The PVA/DMSO solution was poured into a PTFE mold and immersed in glycerol/ethanol (weight ratio of 4/1) solvent mixture for 48 h. The organogels were pre‐stretched to different ratios: 50%, 100%, 150%, and 200%, and then wet‐annealed at 120 °C for 0.5 h. Subsequently, Finally, it was subsequently immersed in deionized water to obtain anisotropic hydrogels. The organogel was placed in a beaker containing 1000 mL of deionized water for 48 h of solvent exchange, and the deionized water was changed at least three times a day to ensure that the residues were replaced by water.

### Mechanical Properties Tests

The mechanical properties of the hydrogel were tested by a universal testing machine (Instron Model 3367, USA) with the tensile speed of 50 mm min^−1^. Notched and un‐notched rectangular hydrogels were used for the pure shear tests to measure the tearing energy.^[^ ^21^, ^62^, ^63^, ^64^ ^]^ The cross sectional area of an un‐notched specimen is defined as *A* and Δ*L*_c_ is the horizontal coordinate corresponding to the highest point of the force‐displacement curve of the notched specimen. And Γ is calculated by:

(1) $$\Gamma =\frac{W\left(\Delta L_{c}\right)}{A}$$

### Fatigue Tests

Single‐notch specimens were used to determine the fatigue resistance of the hydrogels.^[^ ^65^, ^66^, ^67^ ^]^ To prevent dehydration of hydrogel, all fatigue tests were conducted in a water bath with an initial crack length (*c*_0_) less than one‐fifth the specimen width (*L*_0_). Cyclic tests were conducted on a mechanical stretcher (FULETEST, China). A digital camera (AF4915ZTL, Dino‐Lite) recorded the crack growth (∆*c*) over stretch cycles ∆*N* without relaxation to give the crack growth rate d*c*/d*N*. The strain energy density can be calculated by:^[^ ^68^ ^]^

(2) $$W\;\left(\lambda_{\max},N\right)=\int_{1}^{\lambda_{\max}}\mathrm{Sd}\lambda \;\;$$

The applied energy release rate *G* (maximum applied stretch *λ*_max_) of the notched specimen at the *N* ^th^ cycle is obtained from:^[^ ^69^ ^]^

(3) $$G\;\left(\lambda_{\max},N\right)=\;2k\left(\lambda_{\max}\right)\cdot c\left(N\right)\cdot W\left(\lambda_{\max},N\right)$$

where *k* is a slowly varying function of the applied stretch equal to $(3/\sqrt{\lambda_{\max}}$), *c* and *W* are the crack length and integral area of the *N* ^th^ loading cycle, respectively. *G* was varied by varying the stretch *λ*_max_ to obtain different d*c/*d*N*. The fatigue threshold was determined by linearly extrapolating the d*c*/d*N* data to the x‐axis. The anisotropic hydrogel was observed to display no longitudinal extension of the crack after 10000 fatigue cycles.

### SEM Characterization

The hydrogel samples were subjected to cryogenic immersion in liquid nitrogen for 30 min to be fractured. The gels were subsequently dried using a freeze dryer for 48 h to obtain the PVA aerogels. Their surfaces were then gold‐sprayed to observe their morphology with a scanning electron microscope (SEM) (Zeiss Supra55, Germany).

### LSM Characterization

Fluorescence patterns were studied on a CARL ZEISS confocal laser scanning microscope (LSM700). Fluorescent PVA hydrogels were prepared by adding 0.1% sodium fluorescein as a fluorescent marker to the PVA/DMSO precursor solution under the same fabrication process as the conventional hydrogels. The fluorescent marker was excited at 488 nm excitation wavelength, and the hydrogel was endowed with a green pseudo‐color.

### AFM Characterization

AFM characterization was done in a scanning probe microscope(SPM‐9700HT, SHIMADZU, Japan), using silicon cantilevers with a sharpened tetrahedral tip of 14 nm diameter and a nominal spring constant of 9 N·m^−1^ (OMCL‐AC200TS‐R3, Japan). During testing, the applied mode was phase mode, and applied scanning speed was fixed at 1 Hz.

### Rheological Characterization

The dynamic rheological properties of the hydrogels were characterized using a rotational rheometer (DHR, TA Co., USA) equipped with a 25 mm diameter parallel‐plate geometry and a Peltier precise temperature control system. All measurements were conducted under isothermal conditions at 25 °C. The dynamic frequency (ω = 0.1 to 100 rad s^−1^) sweep test was performed at a strain amplitude of 0.1%. The time (t = 0 to 200 s) sweep test was performed with the ω of 6.28 rad s^−1^ and γ of 0.1%.

### X‐Ray Scattering Characterization

The crystalline structure of hydrogels was investigated using Regular X‐ray diffraction (XRD, MiniFex600, Japan). The scan range and rate were 5°‐60° and 10° min^−1^, respectively. The estimated size *D* of crystalline domains could be calculated using the Debye‐Scherrer formula:^[^ ^70^ ^]^

(4) $$D=\frac{k_{0}\lambda_{0}}{\beta \cos\theta }$$

where dimensionless shape factor (k_0_) is set to 1, λ_0_ is 0.154 nm, *β* is the full width at half maximum of the peak.

### Water Content Tests

The hydrogel was dried to constant weight and the initial and dried mass of the hydrogel were noted as m_a_ and m_b_, respectively. Then, the water content can be calculated by:

(5) $$\phi_{\mathrm{water}}=\frac{m_{a}-m_{b}}{m_{a}}\;\times 100\%\;$$

### Measurement of Crystallinity

Differential Scanning Calorimetry (DSC 8500, Perkin Elme, USA) was used to test the crystallinity of PVA hydrogels. Before the measurement, glutaraldehyde was used to cross‐link the hydrogels, which were subsequently dried. The crystallinity of the dried sample (*X*_dry_) is given by:

(6) $$X_{\mathrm{dry}}=\frac{H_{\mathrm{crystalline}}}{H_{\mathrm{crystalline}}^{0}}\;\times 100\%\;$$

where $H_{\mathrm{crystalline}}^{0}(\;$= 138.6 J g^−1^) is the enthalpy for fusing the PVA with 100 wt.% crystallinity at the equilibrium melting point $T_{m}^{0\;}.$ ^[^ ^71^ ^]^ The crystallinity in the swollen state (*X*_swollen_) is given by:

(7) $$X_{\mathrm{swollen}}=X_{\mathrm{dry}}\;\left(1\;-\;\phi_{\mathrm{water}}\right)\times 100\%$$

where ϕ_water_ (%) is the water content of hydrogels.

### Measurement of WAXS and SAXS

Wide‐angle X‐ray scattering (WAXS) tests and small‐angle X‐ray scattering (SAXS) were performed on a NanoSTAR (Bruker AXS) instrument with an X‐ray wavelength of 0.154 nm, an operating voltage of 50 kV, and a current of 0.6 mA, and the WAXS profiles were collected over a 2*θ* range from 3° to 35°. The distance from the sample to the detector was 1045 mm, the scattering vector (*q*) ranged from 0.007 to 0.123 Å^−1^, and the exposure time was 300 s. The intermolecular spacing, inter‐crystal spacing, and interlamellar spacing of the hydrogels were calculated as:^[^ ^72^, ^73^ ^]^

(8) $$L=\frac{2\pi }{q_{\max}}$$

where *q*_max_ is the critical vector corresponding to the peak intensity.^[^ ^74^ ^]^ The molecular spacing and the layer spacing of the hydrogel can be calculated based on the relevant literature and Equation (8). The 2D scattering images were analyzed with Fit2D software from the European Synchronization Radiation Facility. Orientation (∏) can be calculated from the azimuthally integrated intensity distribution curve of the X‐ray scattering pattern:^[^ ^75^ ^]^

(9) $$\prod =\frac{180\;-\;\mathrm{FWHM}}{180}\;$$

where full width at half maximum (FWHM) is the width at half‐maximum of the azimuthal distribution curve along the equatorial reflection.

### Fourier Transform Infrared Spectra Characterization

Fourier transform infrared spectra (Cary610/670, Varian, USA) was used to discuss hydrogen bonding in hydrogels.

### Transmittance Characterization

UV‐visible‐near infrared absorption spectrometer (Carry 5000, Varian, USA) was used to test the transmittance of different hydrogels.

### Cytotoxicity Assays of AH‐150

Mouse embryo fibroblast cells (NIH/3T3) were cultured in DMEM supplemented with 10.0% (V/V) FBS and 1.0% (V/V) penicillin/streptomycin. The cell culture dish was maintained in a 37 °C incubator with 5.0% CO_2_ humidified air.

CCK‐8 method was applied to qualitatively evaluate the cytotoxicity of AH‐150. The proliferation rate of NIH/3T3 cells was quantitatively evaluated by adding CCK‐8 solution at desired time intervals. First, 300 µL of NIH/3T3 cells (density = 1.0 × 10^5^ cells mL^−1^) and 1.0 cm^2^ of AH‐150 were implanted in the 48‐well plate and then co‐cultured for 1, 3, and 7 days. Afterward, the medium was replaced with CCK‐8 solution and cultured for another 0.75 h. The absorbance (OD) of supernatant at 450 nm was measured by a microplate reader (Model 550, Bio‐Rad, Hercules, CA, USA), and the proliferation ability of NIH/3T3 cells was calculated by:^[^ ^27^ ^]^

(10) $$\mathrm{Cell}\;\mathrm{viability}=\frac{OD_{\mathrm{sample}\;}-\;OD_{\mathrm{blank}}}{OD_{\mathrm{control}}\;-\;OD_{\mathrm{blank}}}\;\times 100\%$$

where *OD*_blank_, *OD*_control_, and *OD*_sample_ are absorbance values of solutions in the blank, control, and NM‐treated groups, respectively. At least three samples for each group were conducted and the results represented as mean ± SD.

The cytotoxicity of AH‐150 was further evaluated by the LIVE/DEAD staining method. 1.0 mL of NIH/3T3 cells (density = 5.0 × 10^4^ cells mL^−1^) were implanted in a 6‐well plate and co‐cultured with AH‐150 for 1, 3, and 7 days. The LIVE/DEAD staining method observed cell morphology in these two conditions. Finally, the cells were stained with a mixture of 1.0 µm of Calcein‐AM and 1.0 µm of propidium iodide (PI). Image collection was performed using an inverted fluorescence microscope.

### Cell Fluorescent Staining

The ability of AH‐150 to promote oriented growth of cells was evaluated by the fluorescent staining method. NIH/3T3 (1.0 mL) cells (density = 1.0 × 10^4^ cells mL^−1^) were implanted in a 6‐well plate and co‐cultured with AH‐150 for 24 h. Finally, the cells were stained with a mixture of 1.0 µm of Calcein‐AM and 1.0 µm of propidium iodide (PI). Image collection was performed on an inverted fluorescence microscope. Orientation angles of NIH/3T3 cells were counted quantitatively by Image J software and the average orientation angle measured. At least three samples for each group were performed.

### Tendon Repair Assessment In Vivo

All procedures involving animals were approved by the Institutional Animal Care and Use Committee of Fuzhou University, and the experimental operations were in agreement with the *Guide for the Care and Use of Laboratory Animals* (Ministry of Science and Technology of China, 2006). The animal experimental design was reviewed and approved by the Institutional Animal Care and Use Committee of Fujian Provincial Hospital (the ethical review acceptance number: IACUC – FPH – SL – 20 231 020[0164]). Female SD rats (6−8 weeks old) were purchased from Shanghai SLAC Laboratory Animal Co., Ltd (Shanghai, China) and were kept at 25 °C.

To investigate tendon repair in vivo, SD (Sprague‐Dawley) rats were randomly divided into two groups of Control and AH‐150. They were anesthetized intraperitoneally with 3% pentobarbital sodium, and then the bilateral patellar tendon injury model was created in the rats in vivo. In brief, after hair removal and three times disinfection (with povidone iodine) for bilateral knee joints, the skins were incised to expose the patellar tendons which were cut to form the injury. AH‐150 was sutured to the tendon defect by 5‐0 PDS surgical suture and then skin wounds were sutured and disinfected. The legs of the rats were immobilized with bandages for one week after the surgery. At the 4^th^ week and the 8^th^ week after surgery, the rats were euthanized for subsequent evaluation.

### Gross Morphological Evaluation

At the 4^th^ week and the 8^th^ week, SD rats were euthanized and gross morphology of each sample was evaluated. Macroscopic score was considered considering the signs of inflammation, tendon adhesion, defect filling, shape, and color. These scores of the tendons were blindly performed by an independent evaluator. Then, the thickness and width of tendons were measured by a digital vernier caliper.

### Histology

Tissue sectioning was conducted with a pathology slicer (Leica). Hematoxylin‐Eosin (H&E) staining and Masson staining were conducted to evaluate the morphological organization of tendon in different phases. Processed tissues were fixed with 4% paraformaldehyde for 48 h and then were embedded in paraffin. Conventional paraffin‐embedded tissue sections with thickness of ≈5.0 µm underwent dewaxing, hydration, staining, and dehydration. Image collection was done by using an Upright optical microscope (Nikon, Eclipse E100).

### Immunofluorescence Staining

Sections were immunohistochemistry stained with COL1 (Servicebio), COL3 (Servicebio), and nucleus (Servicebio) to evaluate collagen regeneration. In brief, COL1 was stained red, COL3 green, and nucleus blue. Image collection was conducted on an Upright optical microscope (Nikon, Eclipse E100).

### Histological Evaluation Scoring

Histological evaluation of H&E staining images was conducted using the modified Mankin's score as described previously. The histological evaluation score system (Table S1, Supporting Information) includes four parameters: 1) fiber structure, 2) fiber arrangement, 3) nuclei roundness, and 4) cell density. Each variable was valued 0 to 3 points, with 0 being normal and 3 being maximally abnormal. Histological evaluation scoring of H&E staining image was blindly performed by an independent evaluator.^[^ ^76^ ^]^

## Conflict of Interest

The authors declare no conflict of interest.

## Supporting information



Supporting Information

## Data Availability

The data that support the findings of this study are available from the corresponding author upon reasonable request.
