# Supplementary material for: Strong and Fatigue‐Resistant Hydrogels via Poor Solvent Evaporation Assisted Hot‐Stretching for Tendon Repair
Source: Adv Sci (Weinh). 2025 May 8;12(28):2503697. doi: 10.1002/advs.202503697 (PMC12302550; doi:10.1002/advs.202503697)
Supplement: Supplementary file 1 — Supporting Information [file ADVS-12-2503697-s001.docx]

Supporting Information

**Strong and Fatigue-Resistant Hydrogels via Poor Solvent Evaporation Assisted Hot-Stretching for Tendon Repair**

Huamin Li^a, 1^, Ying Zhang^b, 1^, Haidi Wu^a^, Zhanqi Liu^a^, Cheng Guan^a^, Jin Zhang^*c, d^, Jingyi Chen^e^, Shaohua He^e^, Xuewu Huang^a^, Wancheng Gu^a^, Yiu Wing Mai^f^, Jiefeng Gao^*a^

*^a^* *School of Chemistry and Chemical Engineering, Yangzhou University, No 180, Road Siwangting, Yangzhou, Jiangsu, 225002, China*

*^b^ MOE Key Laboratory for Analytical Science of Food Safety and Biology, College of Chemistry, Fuzhou University, 2 Xueyuan Road, Fuzhou 350108, P. R. China*

*^c^ College of Chemical Engineering, Fuzhou University, 2 Xueyuan Road, Fuzhou 350108, P. R. China*

*^d^ Qingyuan Innovation Laboratory, 1 Xueyuan Road, Quanzhou 362801, P. R. China*

*^e^ Fuzhou University Affiliated Provincial Hospital, 134 Dongjie Road, Fuzhou, Fujian 350001, P. R. China*

*^f^ Department of Mechanical Engineering, The Hong Kong Polytechnic University, Hung Hom, Kowloon, Hong Kong, 999077, P. R. China*

^1^ These authors contribute equally to this article and should be considered as co-first authors.

*Corresponding author: E-mail address: J_Zhang929@fzu.edu.cn; jfgao@yzu.edu.cn

**Supplementary Figures**

**Figure S1.** Mass of stretched organogel with a weight ratio of glycerol to ethanol of 4:1 *versus* wet-annealing time.


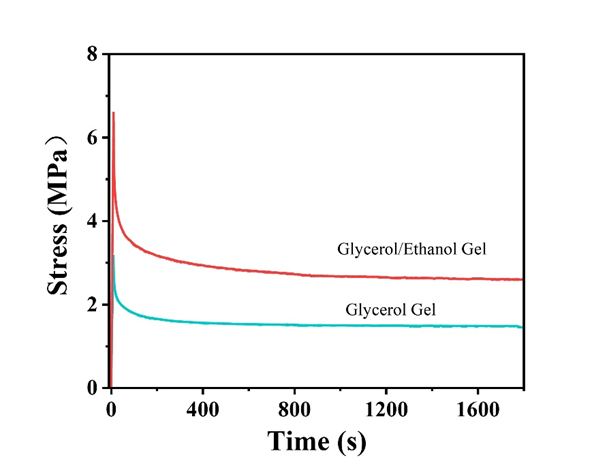


**Figure S2.** The stress-strain curves of the glycerol gel and the glycerol/ethanol gel at a strain of 50% and a temperature of 120 °C.


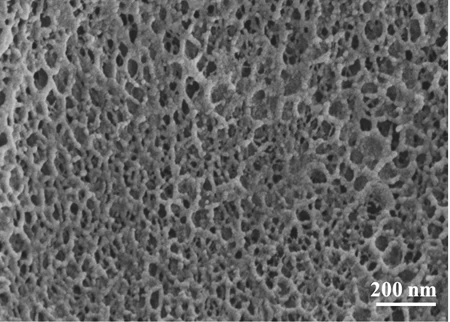


**Figure S3.** SEM image of the cross-section of AH-200 transverse to the orientation direction.


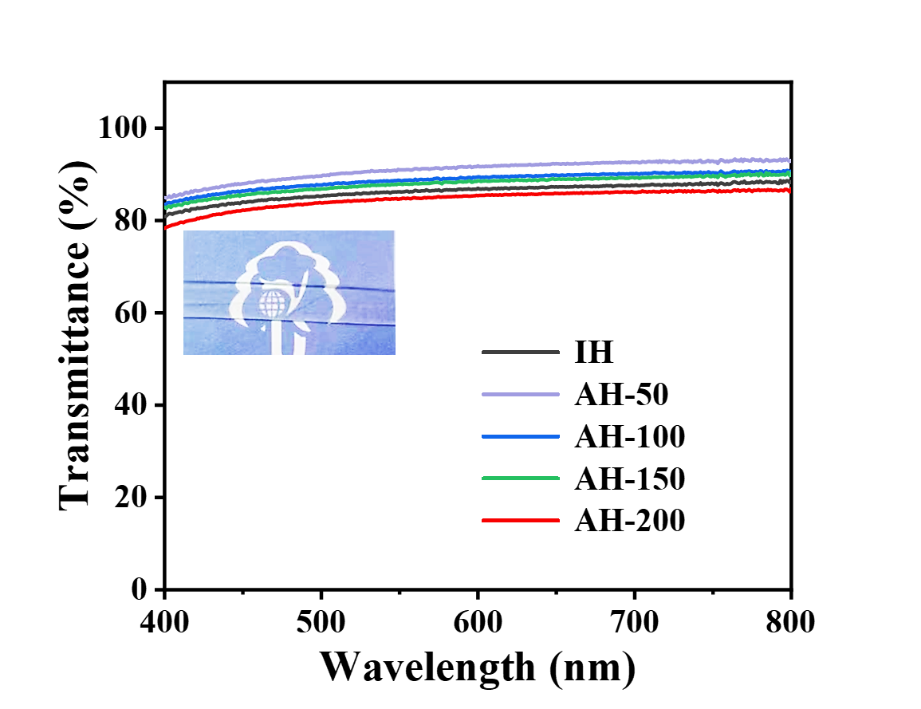


**Figure S4.** Photograph and UV-vis transmittance spectra showing the transparency of the hydrogels.

**Figure S5.** Photograph demonstrating the excellent mechanical strength of AH-200 that could support two dumbbells (total 18 kg) with over 18000x its own weight.


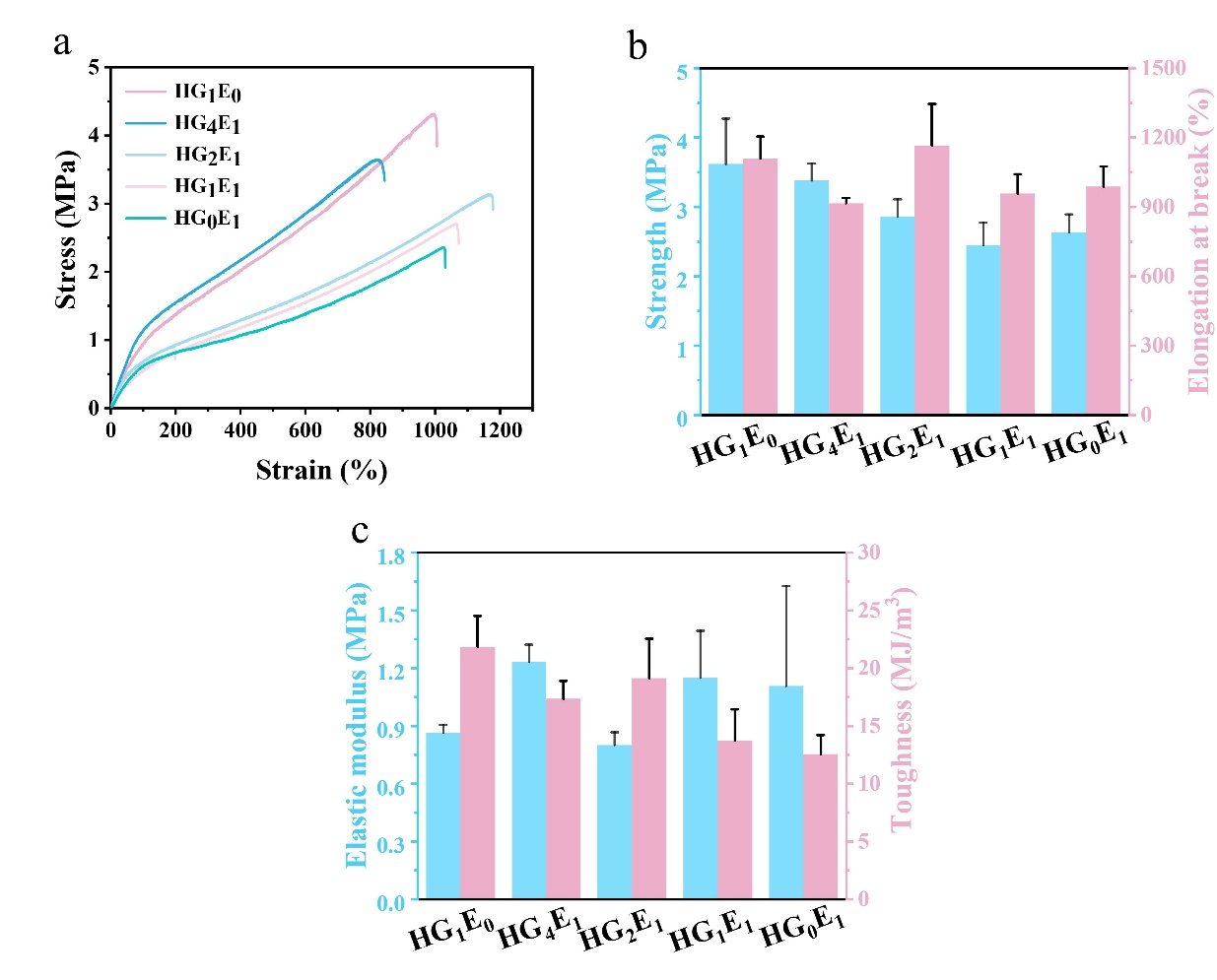


**Figure S6.** Mechanical properties of hydrogels prepared by different glycerol/ethanol ratios. (a) Stress-strain curves, and summary of (b) tensile strength and elongation at break, and (c) elastic modulus and toughness.

**Figure S7.** Water content of hydrogels prepared with different glycerol/ethanol ratios.


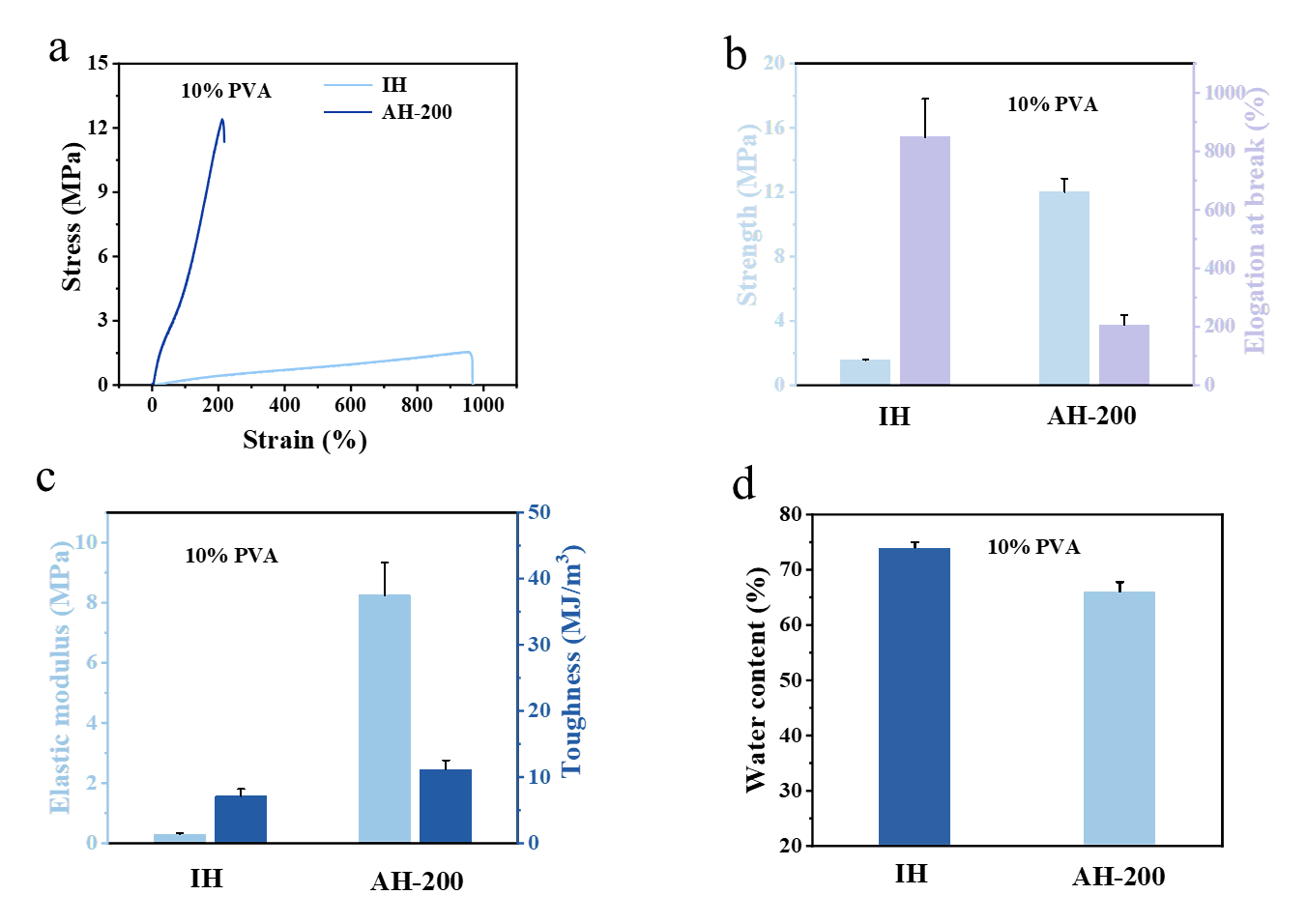


**Figure S8.** (a) Stress-strain curves of isotropic PVA hydrogel (IH) and anisotropic hydrogels (AH-200) prepared with PVA concentration of 10 wt.% and PSR of 200. (b) Tensile strength and elongation at break (10 wt.%). (c) Elastic modulus and toughness (10 wt.%). (d) Water content of these hydrogels.

**Figure S9.** Stress-strain curves of IH and AH-100 in parallel and transverse directions.


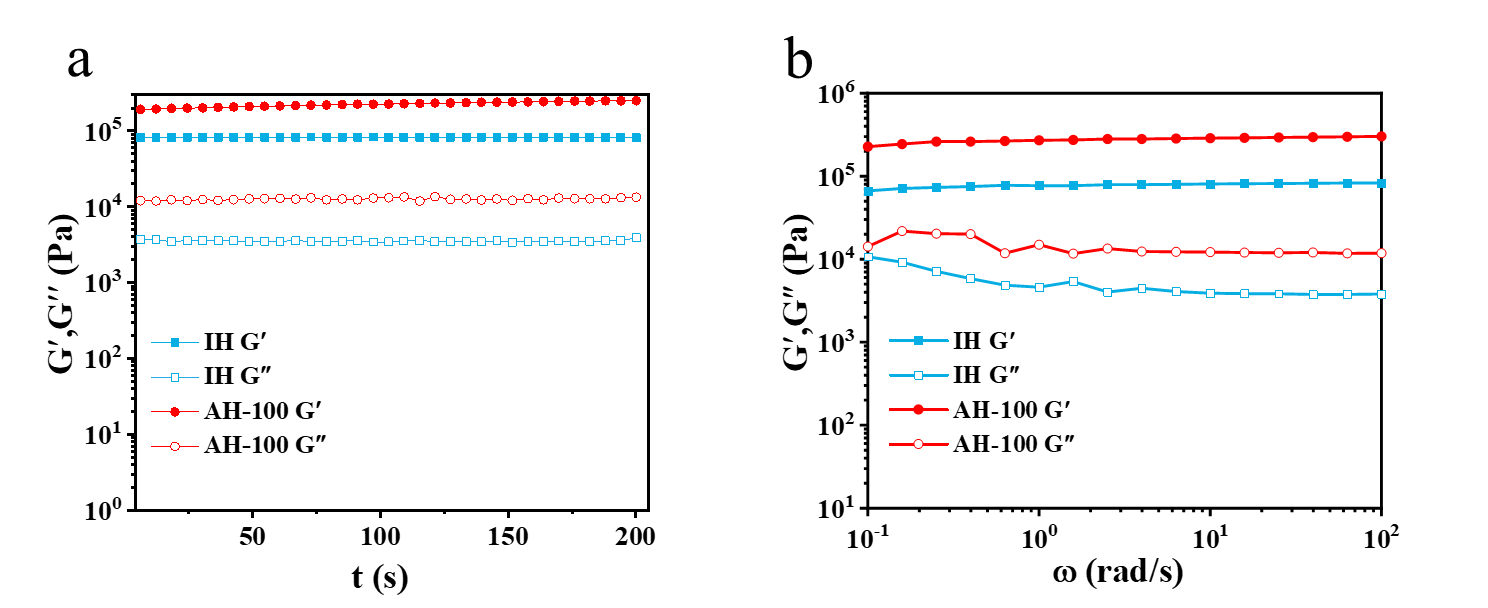


**Figure S10.** Storage modulus (G') and loss modulus (G'') of IH and AH-100 plotted as a function of (a) oscillation time (ω = 6.28 rad s^-1^, γ = 0.1%, T = 25 ℃), and (b) angular frequency (ω = 6.28 rad s^-1^, T = 25 ℃).


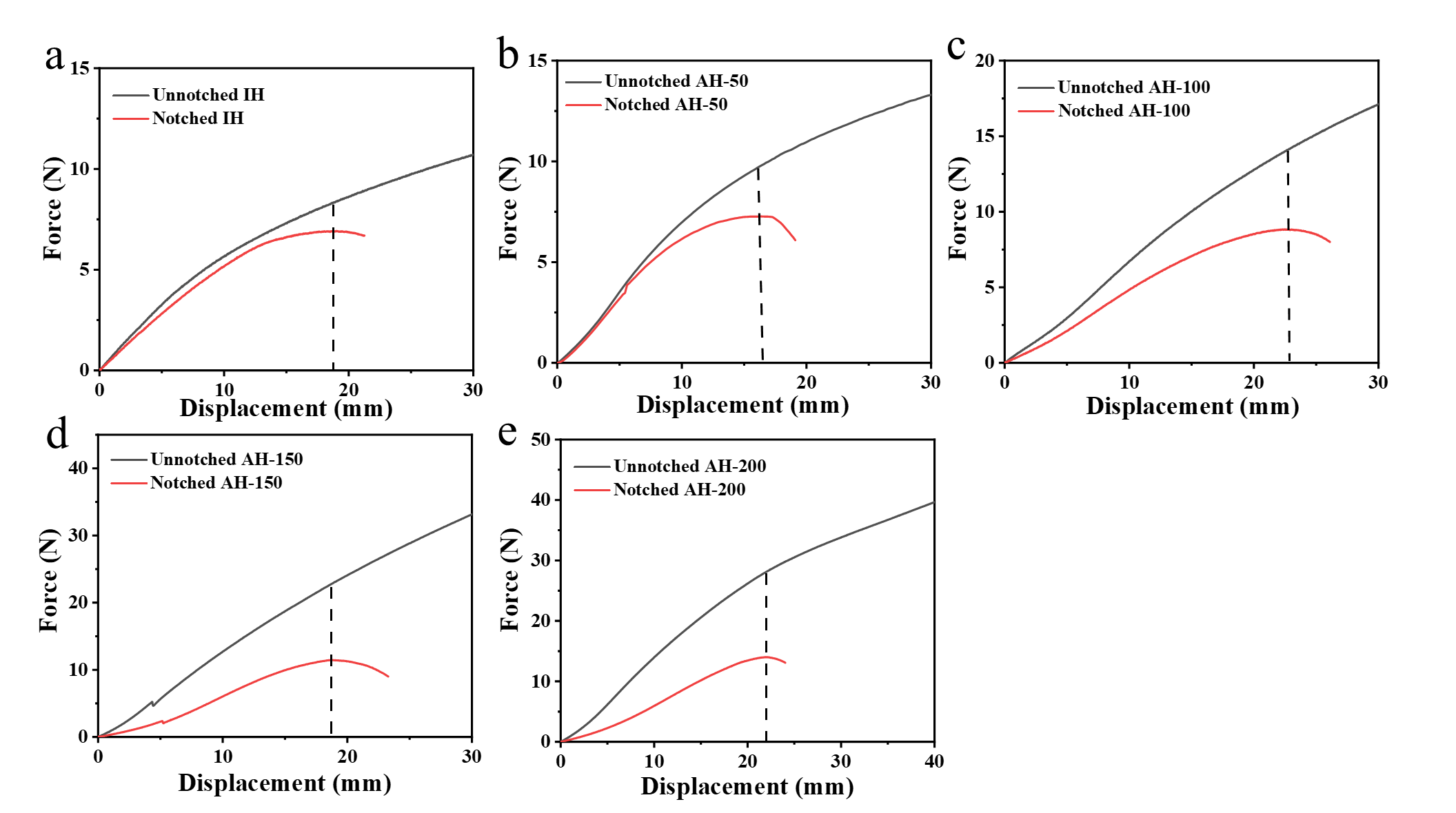


**Figure S11.** The force-displacement curves of unnotched and notched PVA hydrogels. (a) IH, (b) AH-50, (c) AH-100, (d) AH-150 and (e) AH-200.


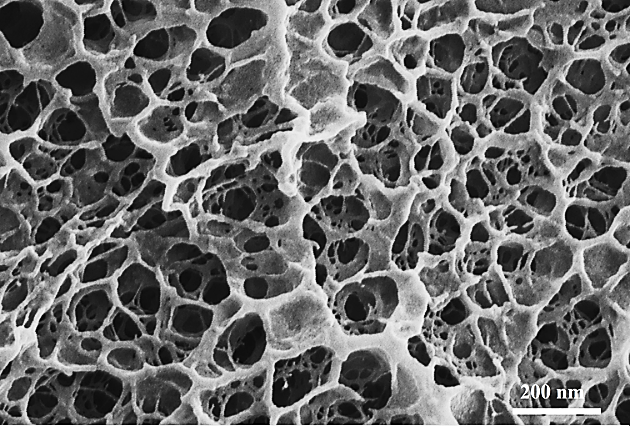


**Figure S12.** SEM image of IH (Pore size = ~256 nm).


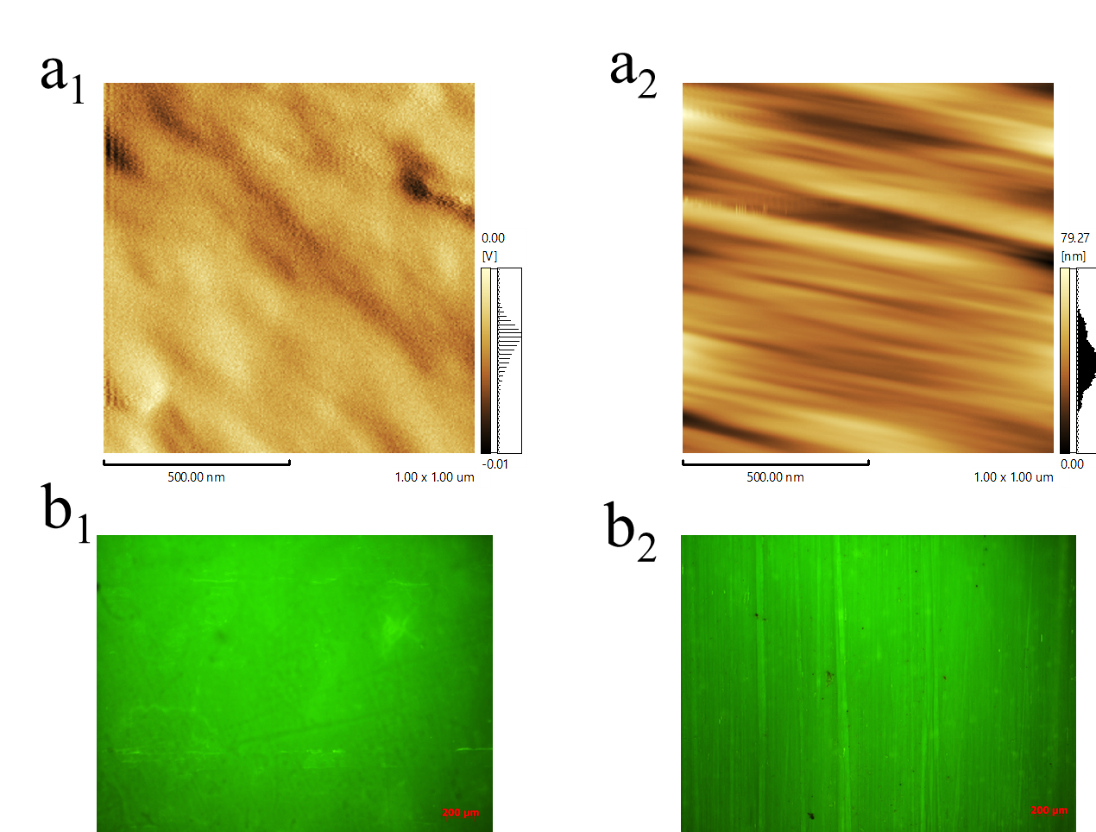


**Figure S13.** AFM images of (a_1_) IH and (a_2_) AH-200. LSM images of (b_1_) IH and (b_2_) AH-200.

**Figure S14.** ATR-FTIR spectra of IH, AH-50, AH-100, AH-150 and AH-200.

**Figure S15.** The orientation degree of IH and AHs calculated from 2D WAXS patterns.


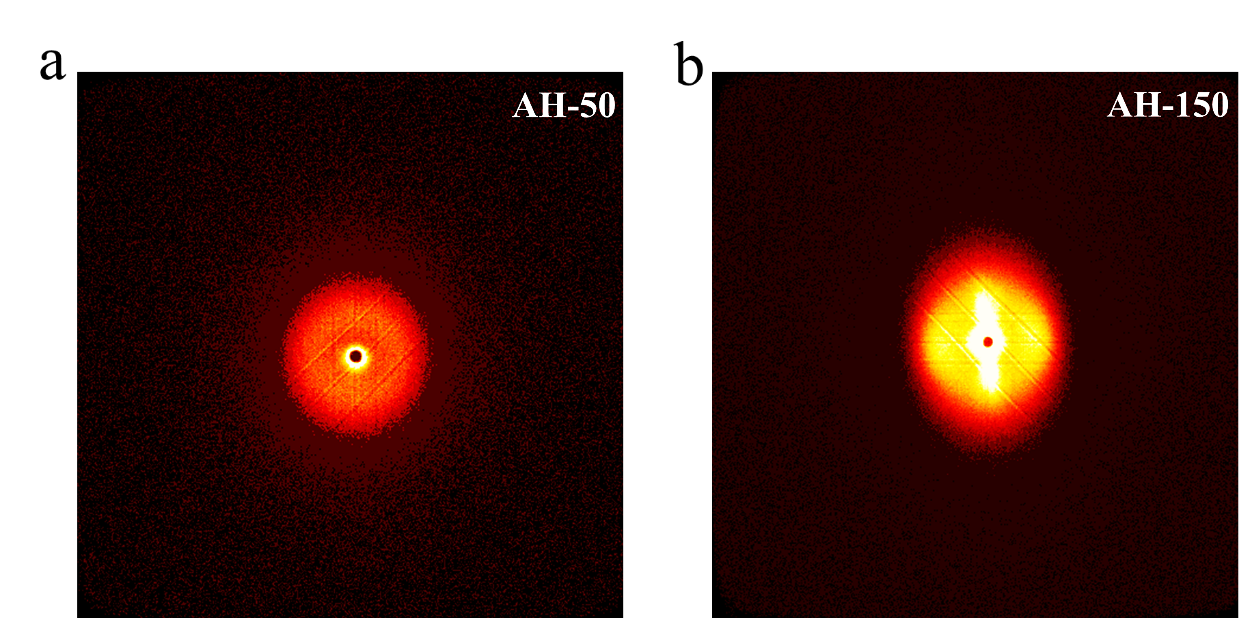


**Figure S16.** 2D SAXS pattern of (a) AH-50 and (b) AH-150.

**Figure S17.** Correlation of the azimuthally integrated intensity distribution of 2D SAXS patterns.

**Figure S18.** Orientation degree of IH and AHs calculated from the 2D SAXS patterns.

**Figure S19.** Summary of average size of crystalline domains of IH, AH-100, and AH-200.


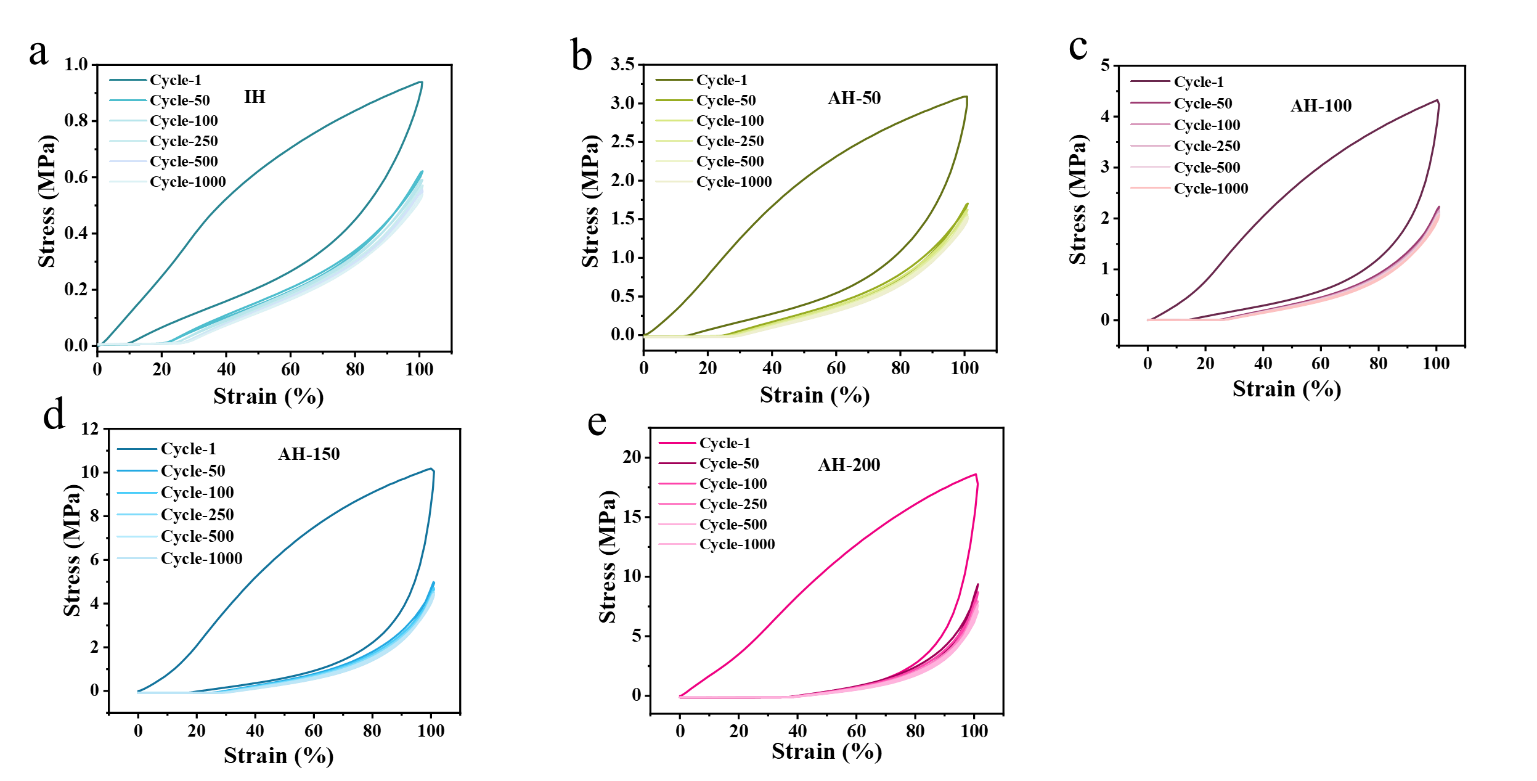


**Figure S20.** (a-e) Stress *versus* strain curves over 1000 cyclic loads for (a) IH, (b) AH-50, (c) AH-100, (d) AH-150 and (e) AH-200.

**
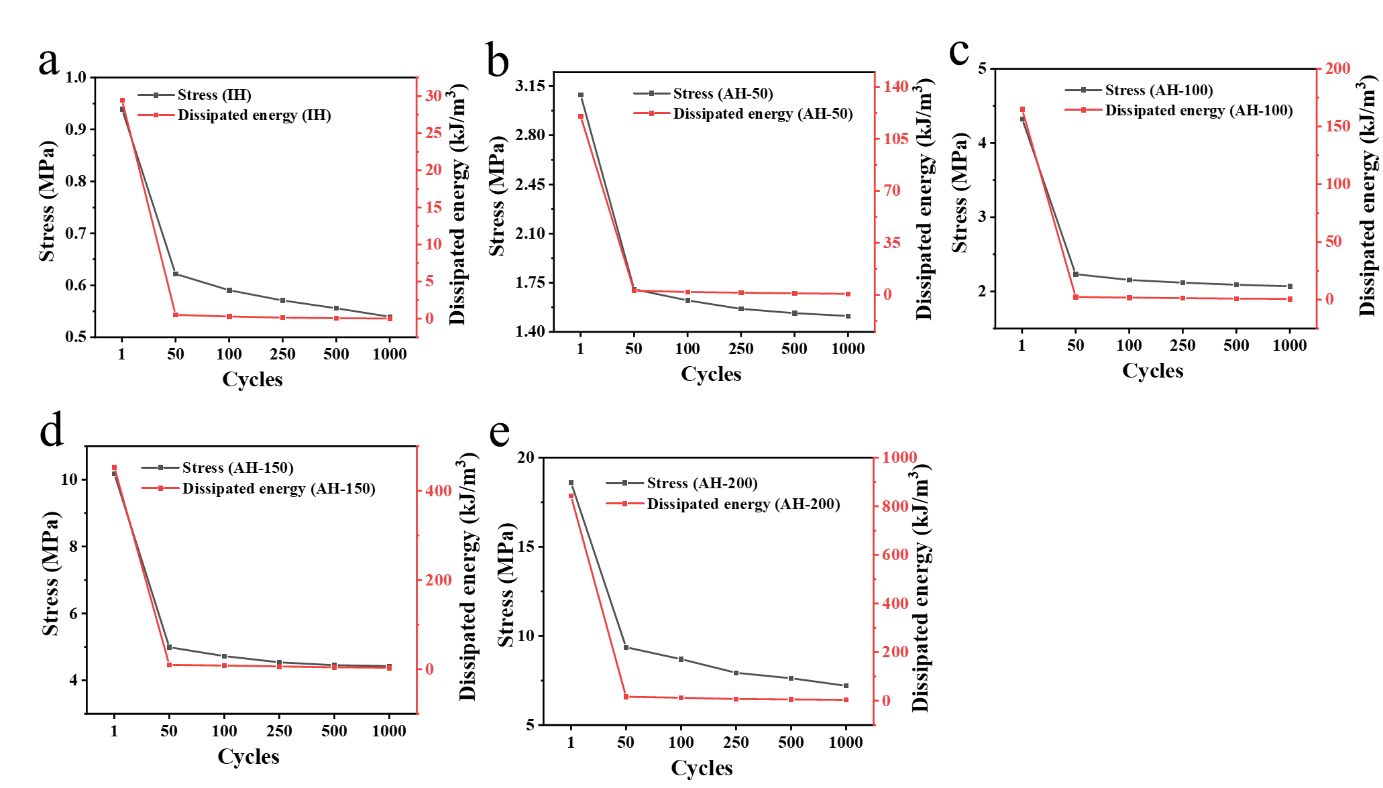
**

**Figure S21.** Stress and dissipated energy during 1000 cyclic loads of (a) IH, (b) AH-50, (c) AH-100, (d) AH-150, and (e) AH-200.

**Figure S22.** Swelling ratio of AH-150 in water, physiological saline and PBS solution over time (3d and 7d).


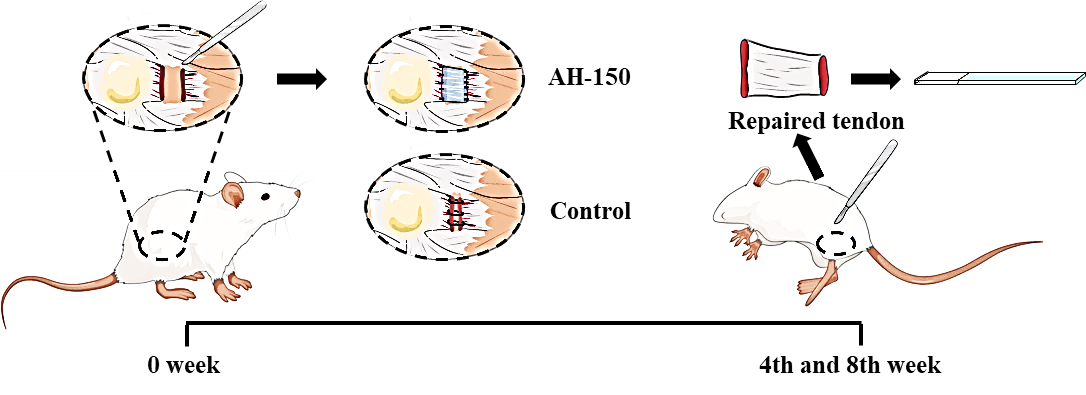


**Figure S23.** Tendon repair experiment in SD rats (Sprague-Dawley rats, 5-6 weeks old, the cartoon was created with Figdraw.com).^[1]^


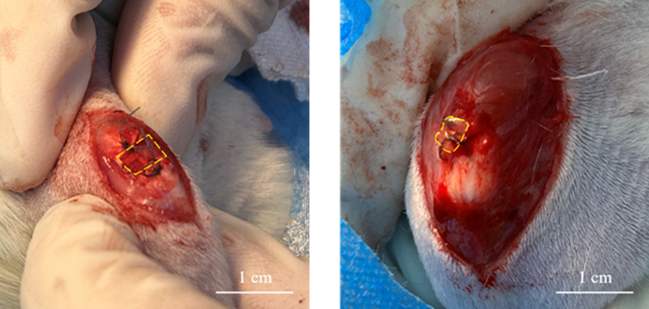


**Figure S24.** Images of AH-150 volume before surgery and 8 weeks after repair, Sale bar: 1 cm.


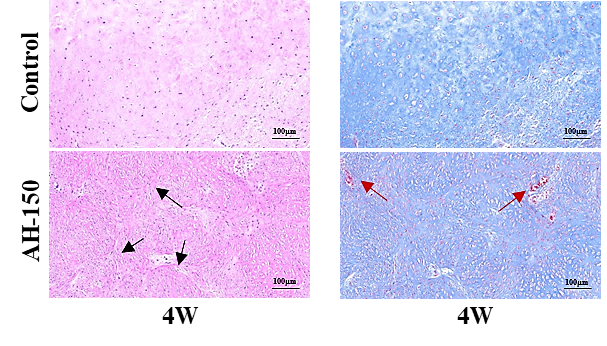


**Figure S25.** H&E and Masson staining of tendon tissues at the 4^th^ week. Black arrows indicate curved fibers and red arrows indicate muscle fibers.


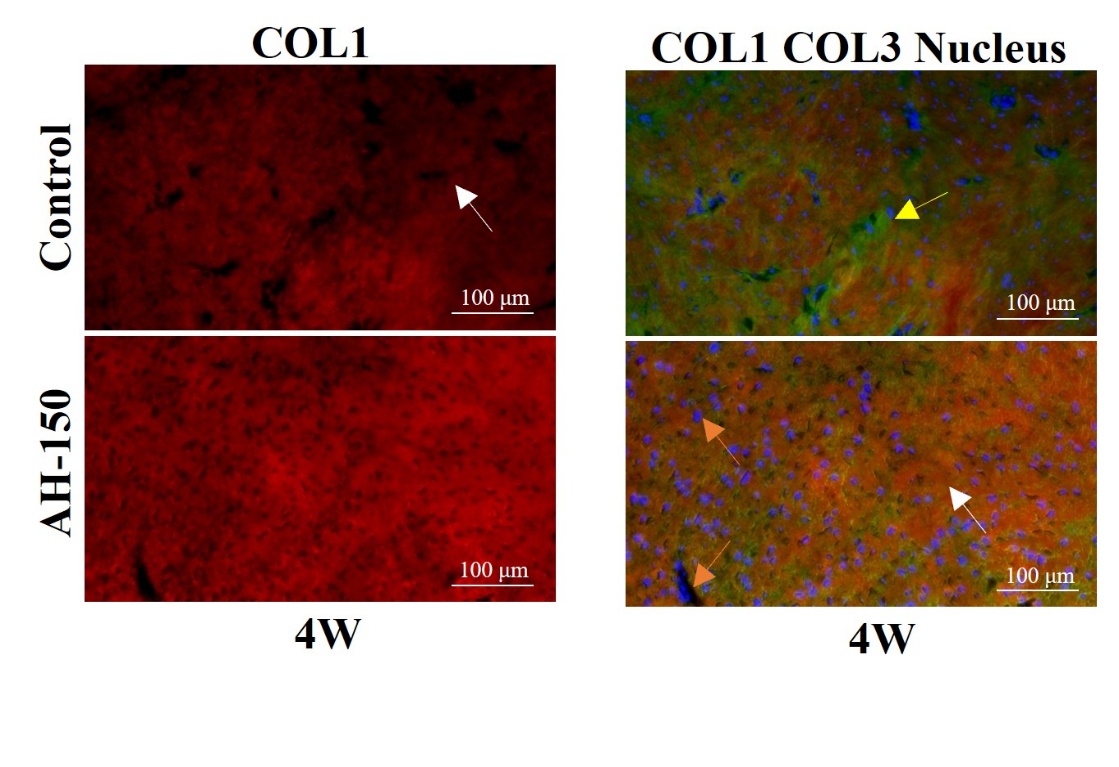


**Figure S26.** Tendon immunofluorescence staining images at 4 weeks.

**
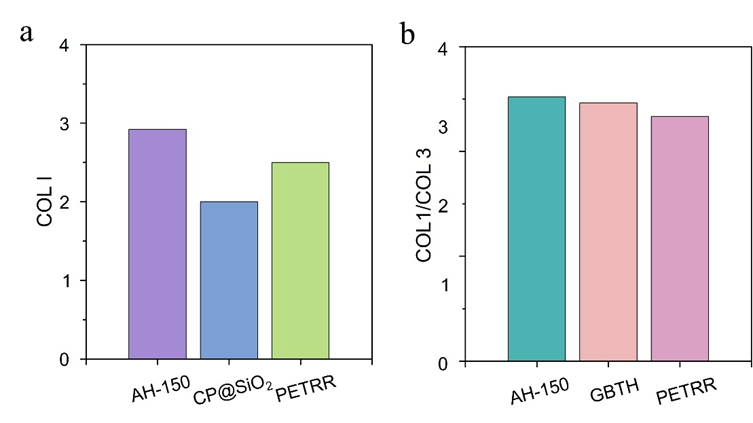
**

**Figure S27.** Comparison of (a) type I collagen (COL1) at 4 weeks and (b) type I/III collagen ratio (COL1/COL3) at 8 weeks in regenerated tendons treated with AH-150 and other hydrogels.

**Table S1.** The histological evaluation score system.

| Tendon repair assessment score | | | | |
| --- | --- | --- | --- | --- |
|  | 0 | 1 | 2 | 3 |
| Fiber structure | Continuous, long fiber | Slightly fragmented | Moderately fragmented | Severely fragmented |
| Fiber arrangement | Compacted and parallel | Slightly loose and wavy | Moderately loose, wavy and cross to each other | No identifiable pattern |
| Uclei roundness | Long spindle shape cells | Slightly round | Moderately round | Severely round |
| Cell density | Normal pattern | Slightly increase | Moderately increase | Severely increase |

This score system was modified from Movin's grading.^[2]^

**References**

[1] X. Yuan, Z. Zhu, P. Xia, Z. Wang, X. Zhao, X. Jiang, T. Wang, Q. Gao, J. Xu, D. Shan, B. Guo, Q. Yao & Y. He, Adv. Sci. **2023**, *10*, 2301665.

[2] T. Movin, A. Gad, F. P. Reinholt & C. Rolf, Acta Orthopaedica **1997**, *68*, 170-175.
